# Structure and function of the N-terminal extension of the formin INF2

**DOI:** 10.1007/s00018-022-04581-y

**Published:** 2022-10-28

**Authors:** Leticia Labat-de-Hoz, Laura Comas, Armando Rubio-Ramos, Javier Casares-Arias, Laura Fernández-Martín, David Pantoja-Uceda, M. Teresa Martín, Leonor Kremer, M. Angeles Jiménez, Isabel Correas, Miguel A. Alonso

**Affiliations:** 1 Centro de Biología Molecular (CBM) Severo Ochoa, Consejo Superior de Investigaciones Científicas and Universidad Autónoma de Madrid, 28049 Madrid, Spain; 2 Instituto de Química Física Rocasolano (IQFR), Consejo Superior de Investigaciones Científicas, 28006 Madrid, Spain; 3 Centro Nacional de Biotecnología (CNB), Consejo Superior de Investigaciones Científicas, 28049 Madrid, Spain; 4 Department of Molecular Biology, Universidad Autónoma de Madrid (UAM), 28049 Madrid, Spain

**Keywords:** INF2, Formins, Calmodulin, Calcium, Actin, NMR

## Abstract

In INF2—a formin linked to inherited renal and neurological disease in humans—the DID is preceded by a short N-terminal extension of unknown structure and function. INF2 activation is achieved by Ca^2+^-dependent association of calmodulin (CaM). Here, we show that the N-terminal extension of INF2 is organized into two α-helices, the first of which is necessary to maintain the perinuclear F-actin ring and normal cytosolic F-actin content. Biochemical assays indicated that this helix interacts directly with CaM and contains the sole CaM-binding site (CaMBS) detected in INF2. The residues W11, L14 and L18 of INF2, arranged as a 1-4-8 motif, were identified as the most important residues for the binding, W11 being the most critical of the three. This motif is conserved in vertebrate INF2 and in the human population. NMR and biochemical analyses revealed that CaM interacts directly through its C-terminal lobe with the INF2 CaMBS. Unlike control cells, INF2 KO cells lacked the perinuclear F-actin ring, had little cytosolic F-actin content, did not respond to increased Ca^2+^ concentrations by making more F-actin, and maintained the transcriptional cofactor MRTF predominantly in the cytoplasm. Whereas expression of intact INF2 restored all these defects, INF2 with inactivated CaMBS did not. Our study reveals the structure of the N-terminal extension, its interaction with Ca^2+^/CaM, and its function in INF2 activation.

**Supplementary Information:**

The online version contains supplementary material available at 10.1007/s00018-022-04581-y.

## Introduction

Formins are a widespread family of proteins involved in polymerizing monomeric globular actin into linear filamentous actin (F-actin) [1, 2]. Formins possess two characteristic domains: a formin homology (FH) 2 domain, which catalyzes actin polymerization, and an FH1 domain, which binds profilin to provide G-actin to the FH2 domain to form F-actin. Most formins contain a diaphanous inhibitory domain (DID) at the N-terminal region that interacts with a diaphanous autoregulatory domain (DAD) located at the C-terminal region to close the molecule in an inactive state [3]. In the case of the formin mDia1, there is an N-terminal extension to the DID with a domain, called the G domain, that forms a bipartite surface in conjunction with the DID to constitute the GTPase-binding domain (GBD), which is responsible for binding the GTPase Rho in its active GTP-loaded form [4–6]. The binding of Rho to the GBD disrupts the DID–DAD interaction and opens mDia1 in a catalytically active form [4, 5, 7]. However, although other formins also bind Rho-family GTPases, this mechanism of activation is not shared by all DID and DAD-containing formins [8].

INF2 is a DID and DAD-containing formin linked to inherited human disease [9]. Pathogenic INF2 mutations are autosomal dominant and produce focal segmental glomerulosclerosis [10, 11], which causes a partial loss of podocytes that may end in renal failure. Depending on the specific mutation, focal segmental glomerulosclerosis is combined or not with Charcot–Marie–Tooth disease [12], which is a neurological disorder affecting the functioning of the peripheral nerves leading to progressive distal muscle weakness [13]. The overall domain organization of INF2 is similar to that of mDia1, except that it has an N-terminal extension of unknown structure and function that is shorter than, and has no homology with, that of mDia1 [14, 15]. Elevation of intracellular Ca^2+^ levels triggers a rapid remodeling of the actin cytoskeleton in an INF2 expression-dependent manner [16, 17]. This remodeling is mediated by the association of calmodulin (CaM) with INF2 [17, 18], but it was not established whether or not the two proteins interact directly. In this work we have addressed the structure and function of the N-terminal extension of INF2 and found it to be organized into two α-helices, the first of which interacts directly with INF2 and contains the sole CaM-binding site (CaMBS) of the INF2 molecule. Inactivation of this site by the mutation of critical residues abolished the actin polymerization response of INF2 to augmented Ca^2+^ levels. In conclusion, whereas mDia1 is activated by the binding of Rho to the bipartite CaM-binding site at the N-terminal region, the direct binding of Ca^2+^/CaM to the CaMBS in the first α-helix of the N-terminal extension does so for the INF2 molecule.

## Results and discussion

### The N-terminal extension of INF2 is organized into two α-helices

**Fig. 1:**
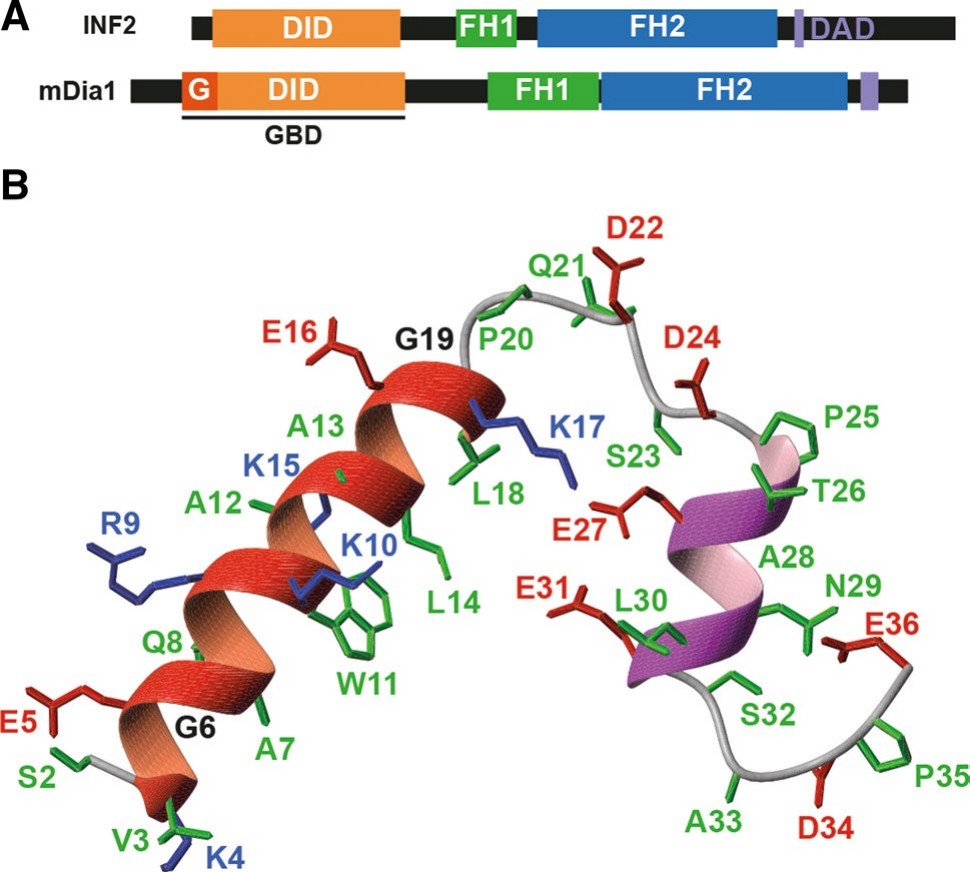
The N-terminal extension of INF2 contains two α-helices. **A** INF2 has an extension N-terminal to the DID that is shorter than that of mDia1 and lacks a G domain. *G* (also known as *GBD*_*N*_) domain necessary for Rho binding that is adjacent to the N terminus of the DID, *GBD* GTPase-binding site, *DAD* diaphanous autoinhibitory domain, *DID* diaphanous inhibitory domain, *FH1 and FH2* formin homology 1 and 2, respectively. **B** Ribbon representation of the NMR solution structure of the N-terminal extension of INF2. Backbone atoms are shown in black. K and R side chains are displayed in blue, D and E side chains in red, and all others in green

Human INF2 has a 35-amino-acid-long extension at the N-terminal end whose structure and function are unknown, while the extension present in mDia1 is longer and contains a part, called the G domain, of the Rho GTPase-binding site (Fig. 1A). The NMR study of a synthetic peptide comprising the full N-terminal extension of INF2 (INF2 2–36) revealed two α-helices, respectively, spanning amino acids 3–17 and the second spanning amino acids 25–31, which were separated by a short unstructured region (Figs. 1B and S1A–D). The two helices were well defined (Fig. S1C, D and Table S1), but not their relative orientation with respect to one another (Fig. S1B). A synthetic peptide comprising only residues 2-19 was shown to form a α-helical structure identical to the first helix of the 2–36 peptide (Fig. S1E, F).

### The first α-helix of the N-terminal extension of INF2 is required for actin homeostasis

**Fig. 2:**
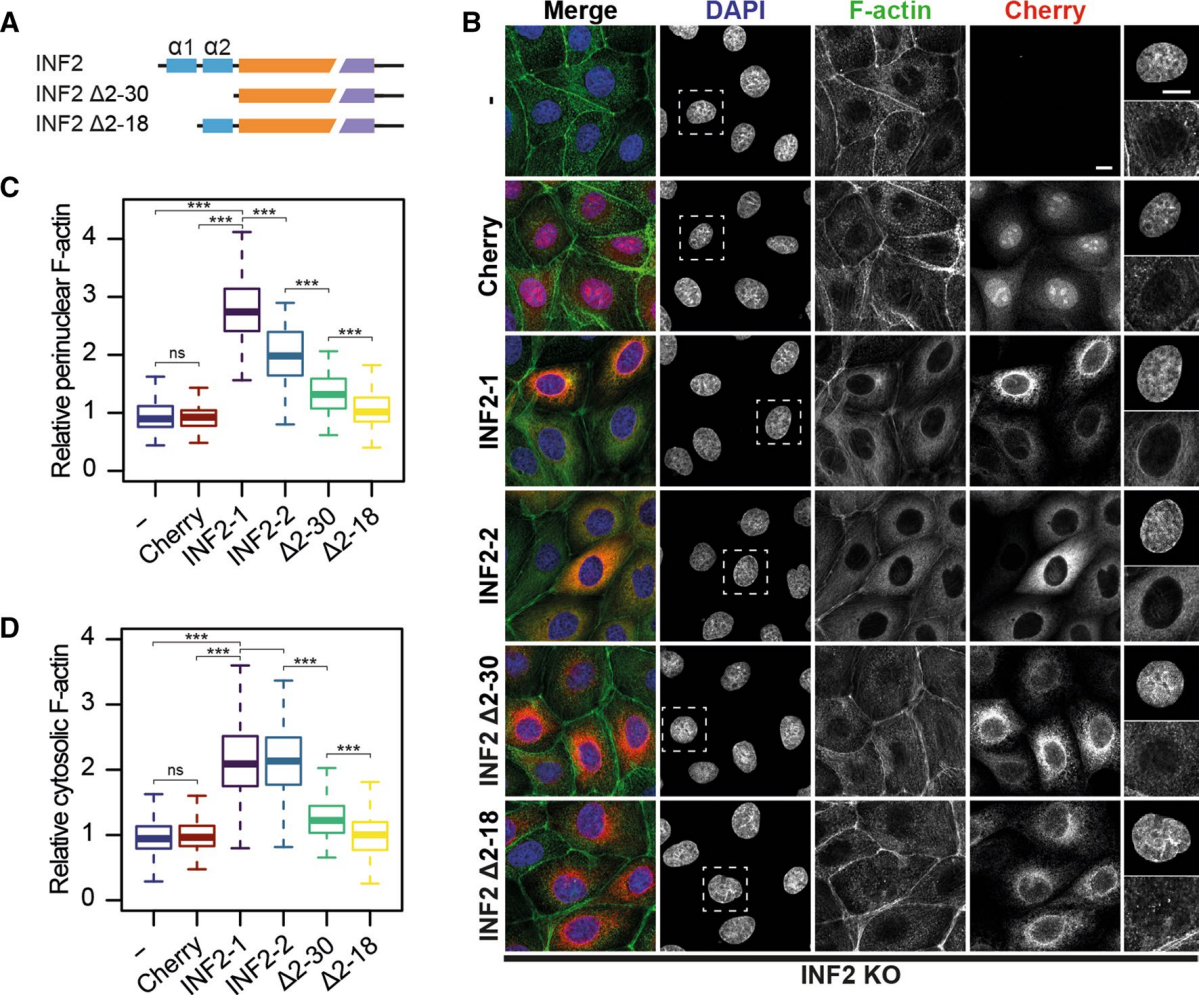
The first of α-helix of INF2 is required for the normal distribution and content of F-actin. **A** Schematic of the INF2 proteins expressed. **B–D** INF2 KO cells or INF2 KO cells expressing mCherry alone or mCherry fusions of the indicated INF2 proteins were stained for F-actin. Nuclei were visualized with DAPI. An enlargement of the DAPI and F-actin staining corresponding to the boxed regions is shown in the rightmost panels. Scale bar, 10 μm (**B**). Box plots showing the intensity of F-actin staining at the perinuclear region (**C**) and the cytosol (**D**) relative to that of control INF2 KO cells (> 140 cells were analyzed for each experimental condition; three independent experiments; *ns* not significant; ***p < 0.001)

For functional studies, we used Madin–Darby canine kidney (MDCK) cells, which are a paradigm of epithelial cells [19]. To identify alterations in the actin cytoskeleton from the lack of INF2 expression, we compared INF2 KO cells [20] expressing the indicated proteins (Fig. 2A). INF2 KO cells lacked the thin ring of F-actin that surrounds the nucleus of MDCK cells under our cell culture conditions [21, 22] and had a lower cytosolic F-actin content than did the control cells (Fig. S2A–C). Consistent with previous findings in HeLa KO cells [17], expression of either of the two isoforms of INF2, INF2-1 and INF2-2 (also known as INF2 CAAX and INF2 nonCAAX, respectively), restored these defects (Fig. 2B–D). However, INF2-1 was more efficient than INF2-2 in polymerizing actin at the perinuclear ring, probably because INF2-1 is associated with the endoplasmic reticulum whereas INF2-2 is cytoplasmic [23]. To analyze the function of the N-terminal extension, we chose INF2-1, hereafter called INF2 for simplicity, to express deletion mutants lacking both helices (INF2 Δ2-30) or only the first of them (INF2 Δ2–18). It is of note that neither of the deletion mutants rescued the normal F-actin phenotype (Fig. 2B–D). The levels of expression in INF2 KO cells of the exogenous INF2 proteins assayed were 6–9 times higher than those of endogenous INF2 in control cells (Fig. S2D, E). No significant differences were found in the expression levels between the exogenous INF2 proteins (Fig. S2D, F). In conclusion, the first α-helix of the N-terminal extension of INF2 is required to ensure the normal distribution and cellular content of F-actin.

### The first α-helix of the N terminus of INF2 binds Ca^2+^/calmodulin

**Fig. 3:**
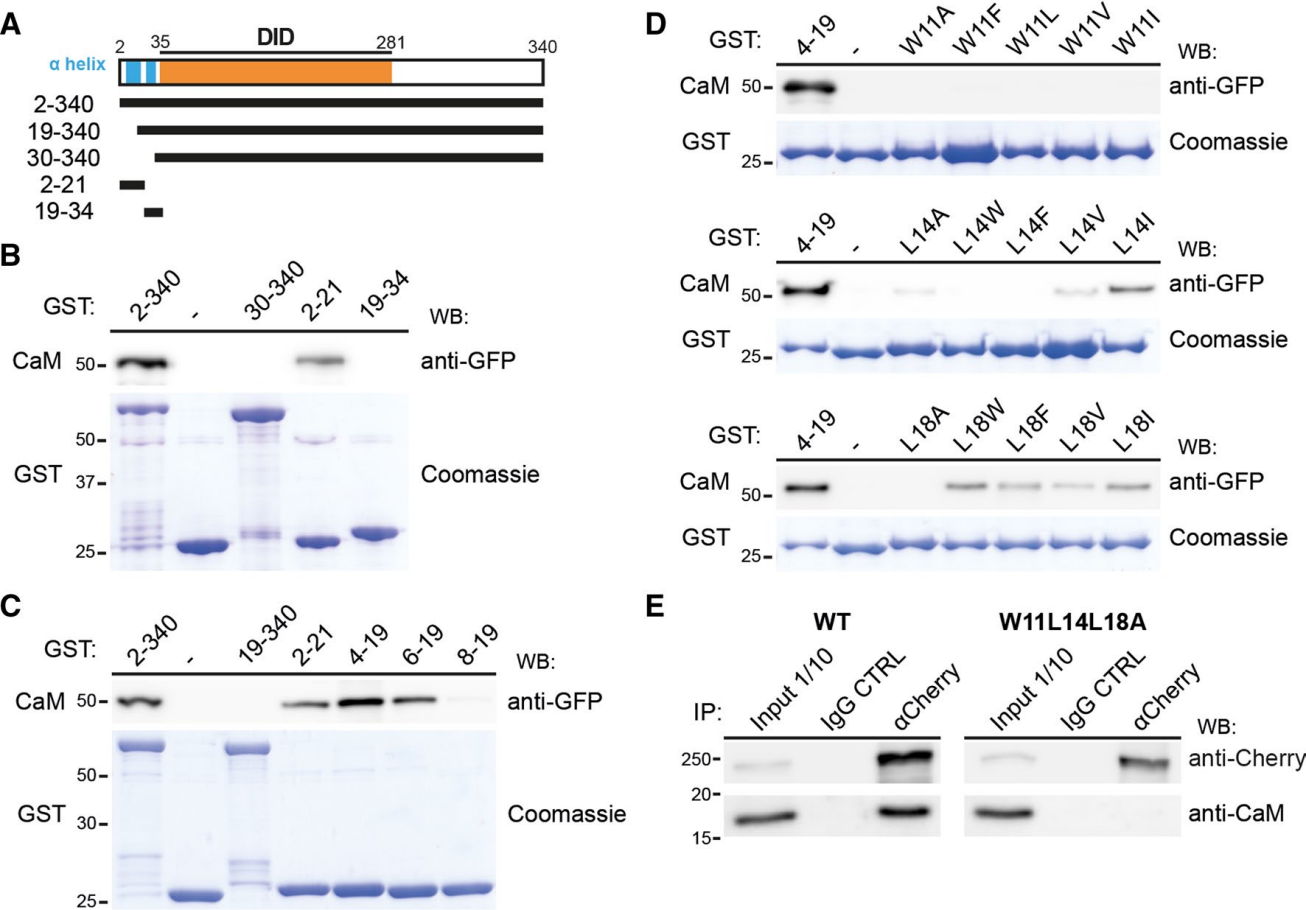
CaM binds the N-terminal α-helix of INF2.** A** Schematic of the INF2 fragments used in the pull-down experiments. **B**, **C** Pull-down analysis of the association of GFP-CaM with GST fusions of the indicated INF2 fragments. **D** GST fusions of the fragment comprising amino acids 4–19 of INF2 with the indicated mutations in residues W11, L14 and L18 were used in pull-down experiments with GFP-CaM. The purified GST fusions were stained with Coomassie blue as a control for the amount of GST fusions used in (**B**–**D**). **E** Lysates of HEK293T cells expressing intact INF2 or INF2 with the triple W11L14L18A mutation fused to mCherry were immunoprecipitated with control or anti-Cherry antibodies and immunoblotted for exogenous INF2 with anti-Cherry antibodies, and with antibodies to endogenous CaM

Elevation of intracellular Ca^2+^ levels triggers a rapid and transient remodeling of F-actin in an INF2 expression-dependent manner in 3T3 fibroblasts [16] and MDCK cells [17]. Since INF2 binds to CaM in the presence, but not in the absence, of Ca^2+^ [17, 18, 24], CaM appears to mediate this response. To investigate whether the actin alterations observed in INF2 KO cells expressing INF2 proteins with N-terminal deletions are correlated with the loss of binding of CaM, we carried out pull-down assays with fragments of the N-terminal region of INF2 fused to GST to analyze GFP-CaM binding (Fig. 3A). We observed Ca^2+^-dependent binding to the INF2 fragment encompassing amino acids 2–340, which contains the DID, the dimerization domain, and the entire N-terminal extension (Fig. S3A). In addition to the 2–340 region, CaM bound to the 2–21 fragment, but not to the fragments 30–340, 19–340 or 19–34 fragments (Fig. 3B, C), indicating that the first α-helix is responsible for the binding of CaM to the 2–340 INF2 fragment. Analysis of sequential deletions of the 2–21 sequence indicated that the 6–19 fragment includes the CaMBS (Fig. 3C).

**Fig. 4:**
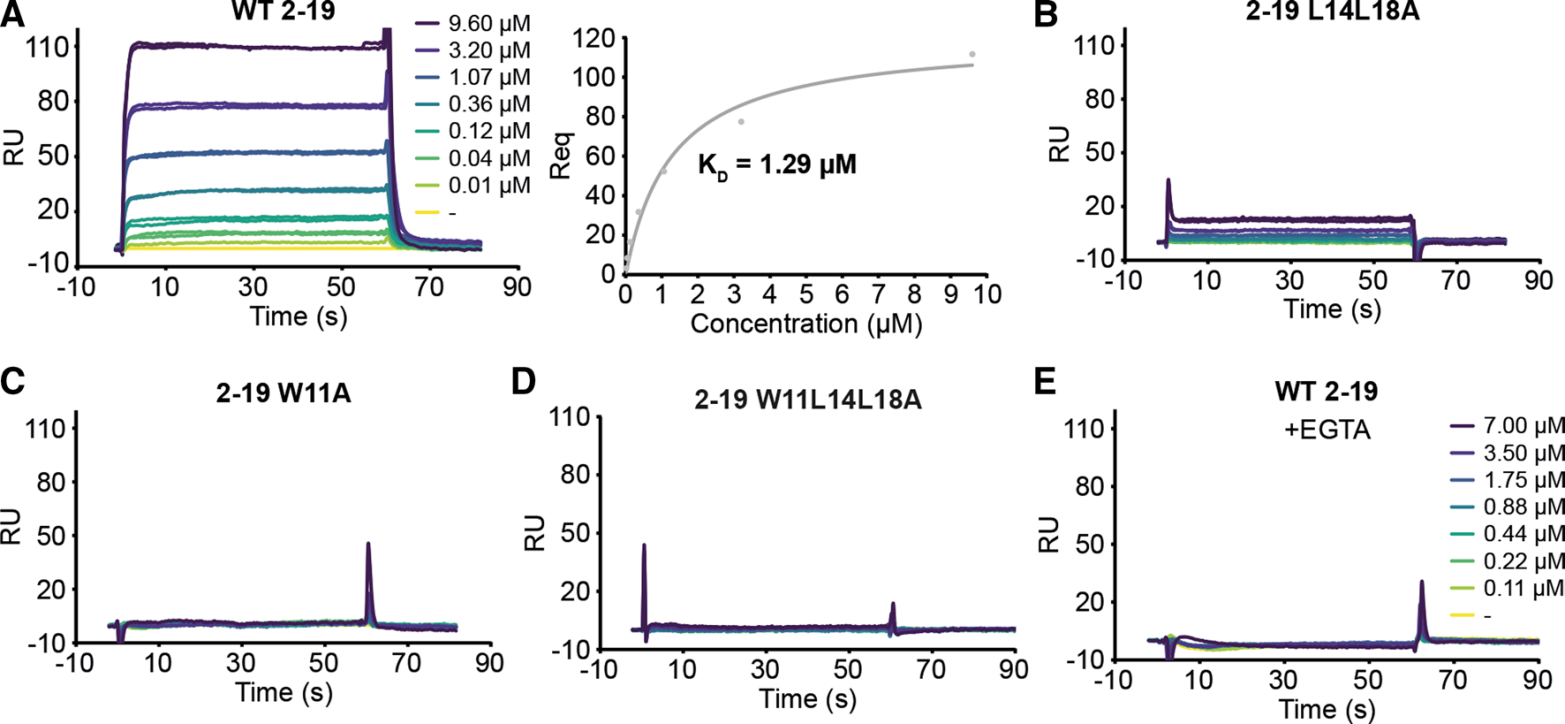
Surface plasmon resonance analysis of the interaction of the 2-19 INF2 peptide with CaM. **A**–**D** Representative equilibrium binding affinity measurements of the interaction of purified CaM from bovine brain with the control 2-19 peptide (A, left panel) or 2-19 peptides containing the L14L18A (B), W11A (C) and W11L14L18A (D) mutations in the presence of Ca^2+^. **E** Control of the binding of CaM to the 2-19 peptide in the absence of Ca^2+^. The equilibrium plots of CaM with the control 2-19 (A, right panel) is shown, with indication of the apparent *K*_D_ resulting from the same experiment. *RU* resonance units, *Req* RU at equilibrium

We carried out a point-mutational analysis of the 6–19 peptide and found residue W11 to be essential for the binding of CaM, since its substitution by A or by any hydrophobic amino acid totally blocked CaM binding (Fig. 3D). Mutation of residues L14 and L18 to A diminished and suppressed, respectively, the binding of CaM. Whereas L18 tolerated substitution by any hydrophobic amino acid, although with reduced binding, the replacement of L14 by W or F, but not by V or I, abolished CaM binding (Fig. 3D). Individual substitution of each of the other residues by A did not significantly alter the binding of CaM (Fig. S3B, C). Mutation of A12 and A13 by Q did not affect the binding of CaM. In the case of A12, the substitution by E did not abolish, but did reduce, CaM binding despite the dramatic change in size and charge (Fig. S3C). The importance of the 2-19 INF2 sequence and the involvement of the three residues—W11, L14 and L18—was confirmed by pull-down assays with GST fused to CaM and cell extracts containing INF2 Δ2-18 or full-length INF2 proteins with single (W11A or L14A), double (L14L18A) or triple (W11L14L18A) mutations (Fig. S3D), and by co-immunoprecipitation analysis of intact INF2 and INF2 W11L14L18A with endogenous CaM (Fig. 3E). Equilibrium binding affinity measurements by surface plasmon resonance analysis of the interaction between a synthetic peptide covering the 2-19 sequence and purified bovine brain CaM yielded an apparent *K*_D_ = 1.40 ± 0.48 μM (mean ± SD; 5 independent measurements; representative experiment in Fig. 4A). A similar value (*K*_D_ = 1.2 ± 0.48 μM; 5 independent measurements; representative experiment in Fig. S3E) was obtained with CaM purified from bovine testes. A longer peptide encompassing amino acids 2–36 yielded a *K*_D_ value in the same range as that of the 2-19 peptide, indicating that the INF2 sequence most proximal to the C-terminal of the 2-19 peptide has no effect on the affinity (Fig. S3F). The result of these experiments, which were done using purified CaM and synthetic peptides corresponding to the N-terminal extension of INF2, indicates that CaM interacts directly with INF2. The double L14L18A 2-19 peptide mutant displayed a reduced affinity with respect to the intact 2-19 peptide (Fig. 4B), whereas the interaction was undetectable for the single W11A and the triple W11L14L18A peptide mutants (Fig. 4C, D) and for the intact peptide in the absence of Ca^2+^ (Fig. 4E). In summary, the results in Figs. 3, 4 and S3 show that the site identified in the first helix of INF2 is the sole CaMBS detected in INF2, and that the affinity of the peptide–CaM interaction is within the low μM range.

### The CaMBS of INF2 binds the C-terminal lobe of CaM, and is conserved in vertebrates and in the human population

**Fig. 5:**
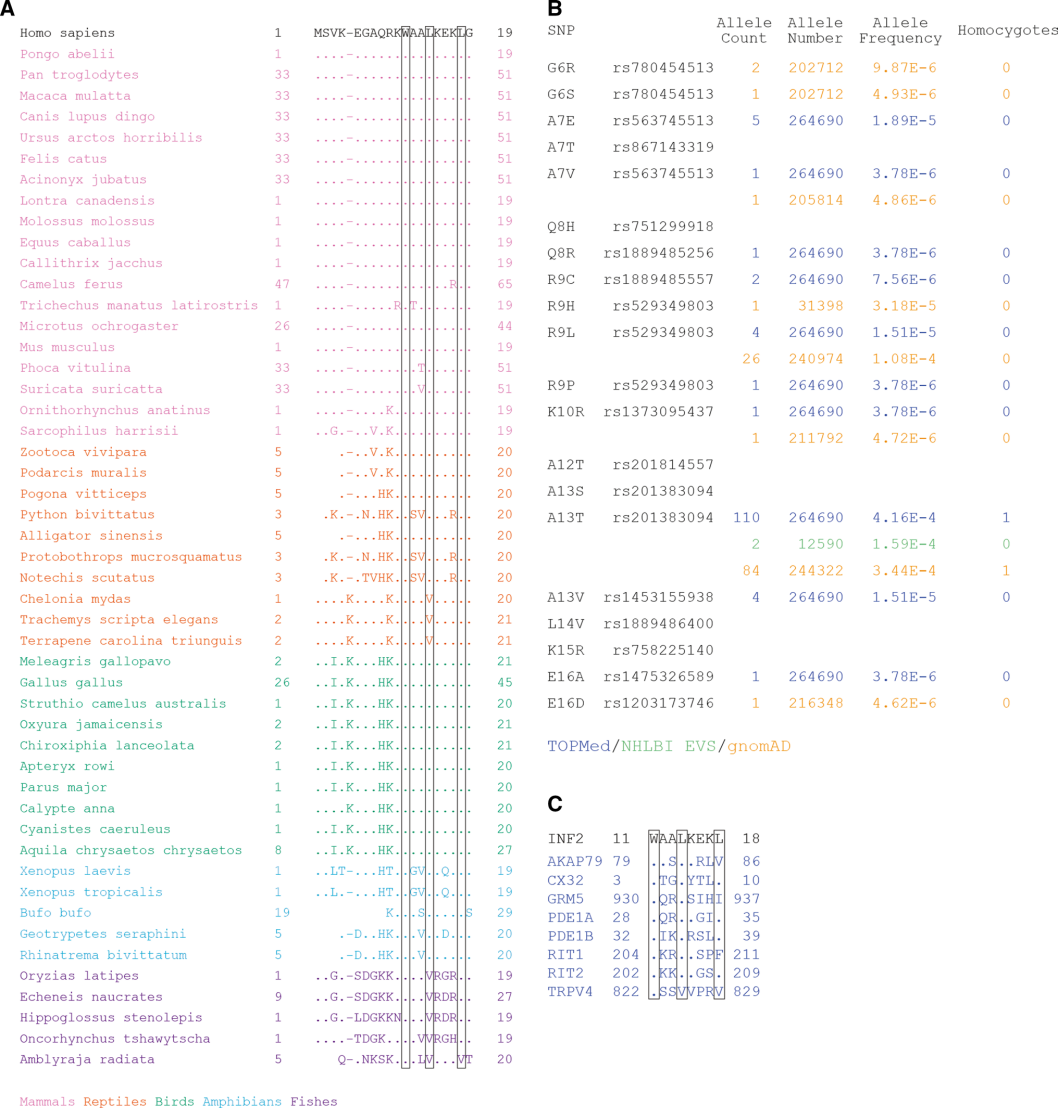
Conservation of the CaMBS of INF2. **A** Alignment of the sequence of the 1–19 peptide of human INF2 with that of 50 species from different groups of vertebrates. The corresponding position of the W11, L14 and L18 residues of human INF2 is indicated. **B** Compilation of allelic variants of the 6–19 sequence of INF2 in the general human population, with indication of the SNP reference, database and frequency. **C** Examples of CaM-binding proteins with a sequence similar to the 1-4-8 motif of INF2. *AKAP79* A-kinase-anchoring protein 79, *CX32* connexin 32, *GRM5* glutamate metabotropic receptor 5, *PDE1A and PDE1B* Ca^2+^/CaM-dependent 3´,5´-cyclic nucleotide phosphodiesterases 1A and 1B, *RIT1 and RIT2* Ras-related GTPases 1 and 2, *TRPV4* transient receptor potential vanilloid subtype 4

Phylogenetic analysis of the human 2-19 INF2 sequence in vertebrates shows the absolute conservation of residue W11 in all the species examined, whereas residues L14 and L18 are also maintained, except in the cases of conservative substitution of L14 by V in some reptiles, and of L14 and L18 by V in fishes (Figs. 5A and S4A). The analysis of the INF2 sequences deposited in public repositories indicated that, whereas most residues in the 6–19 peptide present multiple allelic forms in the human population, residues W11 and L18 are fully preserved and L14 shows only a low-frequency substitution by V (Figs. 5B and S4B). It is of note that all the pathogenic variants of INF2 except A13T are excluded from the N-terminal extension. This mutation, which was initially assigned as a mutation responsible for focal segmental glomerulosclerosis [10], was subsequently considered to be just a polymorphism with no pathological implications, since it is found at a significant frequency in the normal human population [9, 11], and behaves as normal INF2 when it is expressed in cultured cells, unlike the pathogenic INF2 forms, which are constitutively active [18]. In conclusion, these analyses indicate selective preservation of the most critical residues of the INF2 CaMBS.

Canonical motifs for Ca^2^-dependent binding of CaM usually consist of an α-helix with two bulky, hydrophobic amino acids (W, F, I, L, V) at the first and last positions, spaced by a short stretch of a variable number of residues [25]. The motifs involved in Ca^2+^-dependent binding of CaM are classified according to the position of the hydrophobic amino acids involved in the binding [25, 26]. Our mutational and structural analyses indicated that the CaMBS of INF2 corresponds to a 1-4-8 motif in which W11 occupies the position 1 and L14 and L18 the positions 4 and 8, respectively. This type of motif has not been identified before, although it is present in other proteins known to bind CaM (Fig. 5C).

**Fig. 6:**
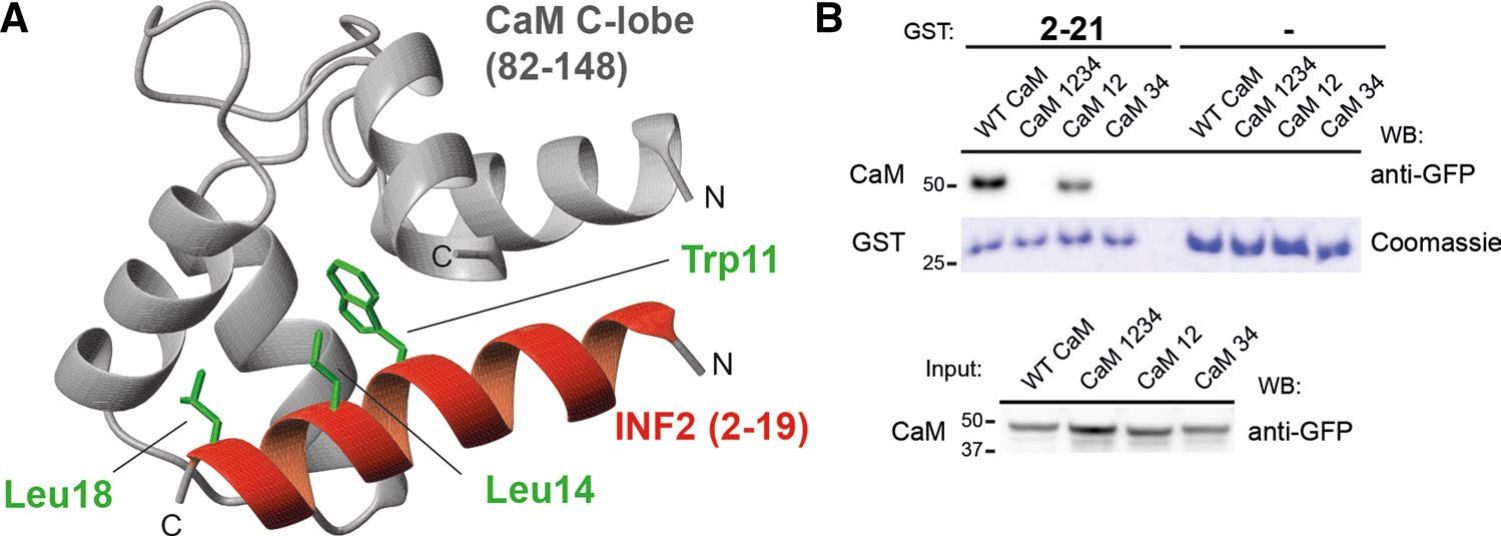
Structural model of the CaM/CaMBS complex. **A** Structural model of C-lobe of CaM bound to the 2-19 INF2 peptide. The position of the three hydrophobic amino acids comprising the 1-4-8 motif of the CaMBS of INF2 is indicated. **B** GST, alone or fused to the 2–21 peptide of INF2, was used in pull-down experiments with GFP fused to intact (WT) or to CaM with mutations that inactivate the two lobes (mutant 1234), the N-terminal lobe (mutant 12) or the C-terminal lobe (mutant 34). The purified GST fusions were stained with Coomassie blue as a control for the amount of GST protein used

CaM contains four canonical EF hands that act as high-affinity Ca^2+^ binding motifs. Two of them are located at the N terminus (N-lobe), and the other two are situated at the C terminus (C-lobe), the two lobes being connected by an extremely flexible central linker region [27]. We used NMR to determine how CaM binds the 2-19 INF2 peptide. Upon titration with the 2-19 INF2 peptide the cross-peaks in the ^1^H,^15^N-HSQC spectra of CaM shifted (Fig. S4C), which confirmed that the 2-19 INF2 peptide binds CaM. The CaM residues experiencing the greatest changes were mainly located at the C-terminal lobe of CaM (Fig. S4D), indicating that the binding to the 2-19 INF2 peptide takes place through this domain. We considered the residues involved in the interaction, i.e., those identified in the 2-19 INF2 peptide, as being important for the binding (Fig. 3D), and those of CaM that underwent the largest chemical shift perturbation (Fig. S4D), and obtained a model of the complex (Fig. 6A) by docking the 2-19 INF2 structure (Fig. S1E) to a CaM structure (pdb code: 3CLN). The INF2 W11 residue appears trapped in a cleft formed by the two EF hands of the CaM C-lobe, which is the canonical binding position for the first hydrophobic anchor amino acid. That the C-terminal lobe of CaM mediates the binding was confirmed by pull-down experiments using GST fused to the INF2 2–21 peptide and GFP-CaM proteins with mutations that inactivated either the EF hands of the N-terminal or the C-terminal lobes, or all the EF hands simultaneously (Fig. 6B).

### The calmodulin-binding site of INF2 is crucial for INF2 activity

**Fig. 7:**
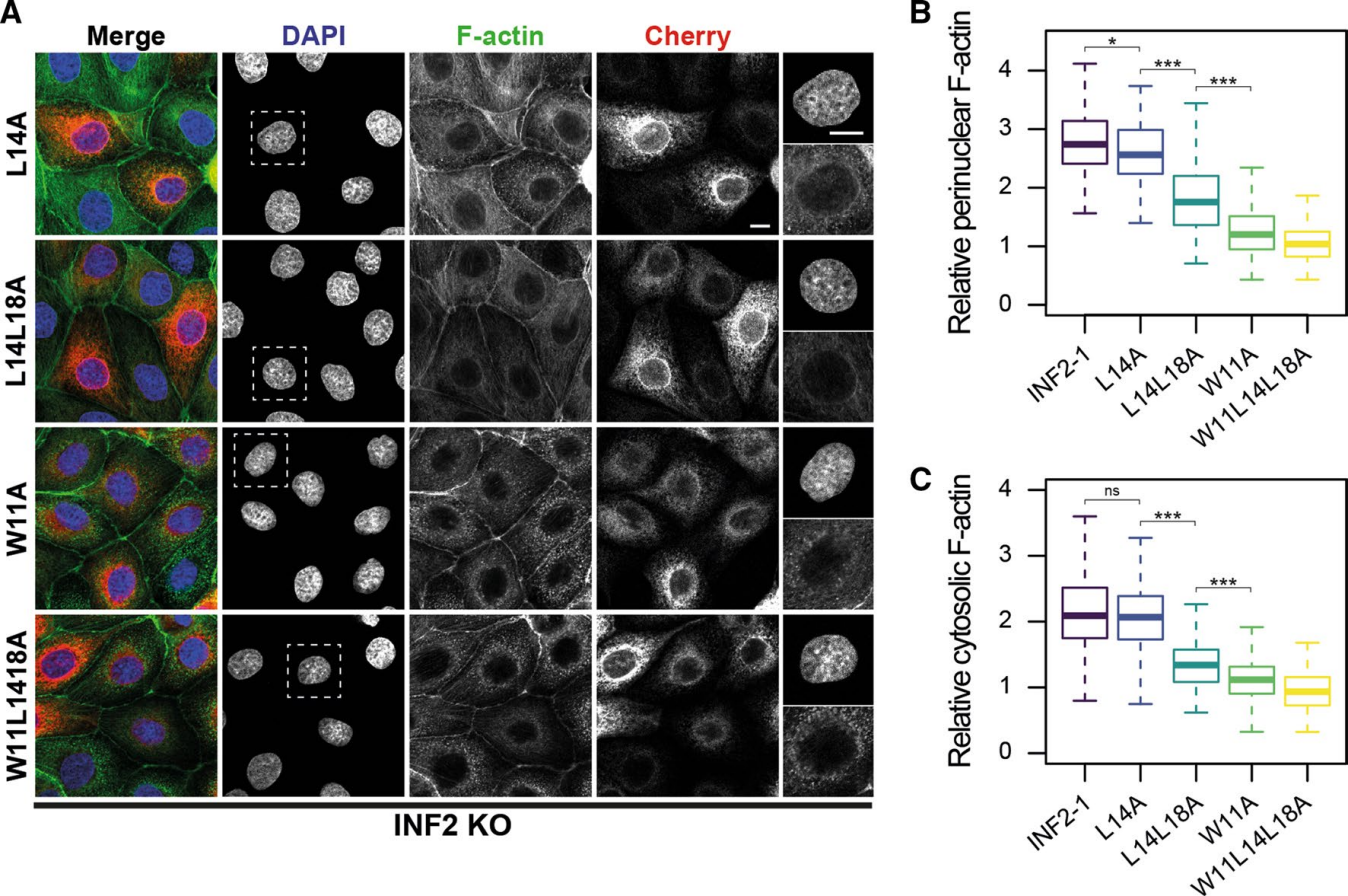
Integrity of the CaMBS of INF2 is crucial for actin homeostasis. **A**–**C** INF2 KO cells expressing mCherry fusions of the indicated INF2 mutants were stained for F-actin. Nuclei were visualized with DAPI. An enlargement of the DAPI and F-actin staining corresponding to the boxed region is shown in the rightmost panels. Scale bar, 10 μm(A). Box plots showing the intensity of F-actin staining at the perinuclear region (**B**) and the cytosol (**C**) relative to that of control INF2 KO cells (> 150 cells were analyzed for each experimental condition; three independent experiments; *ns* not significant; *p > 0.05; ***p < 0.001)

**Fig. 8:**
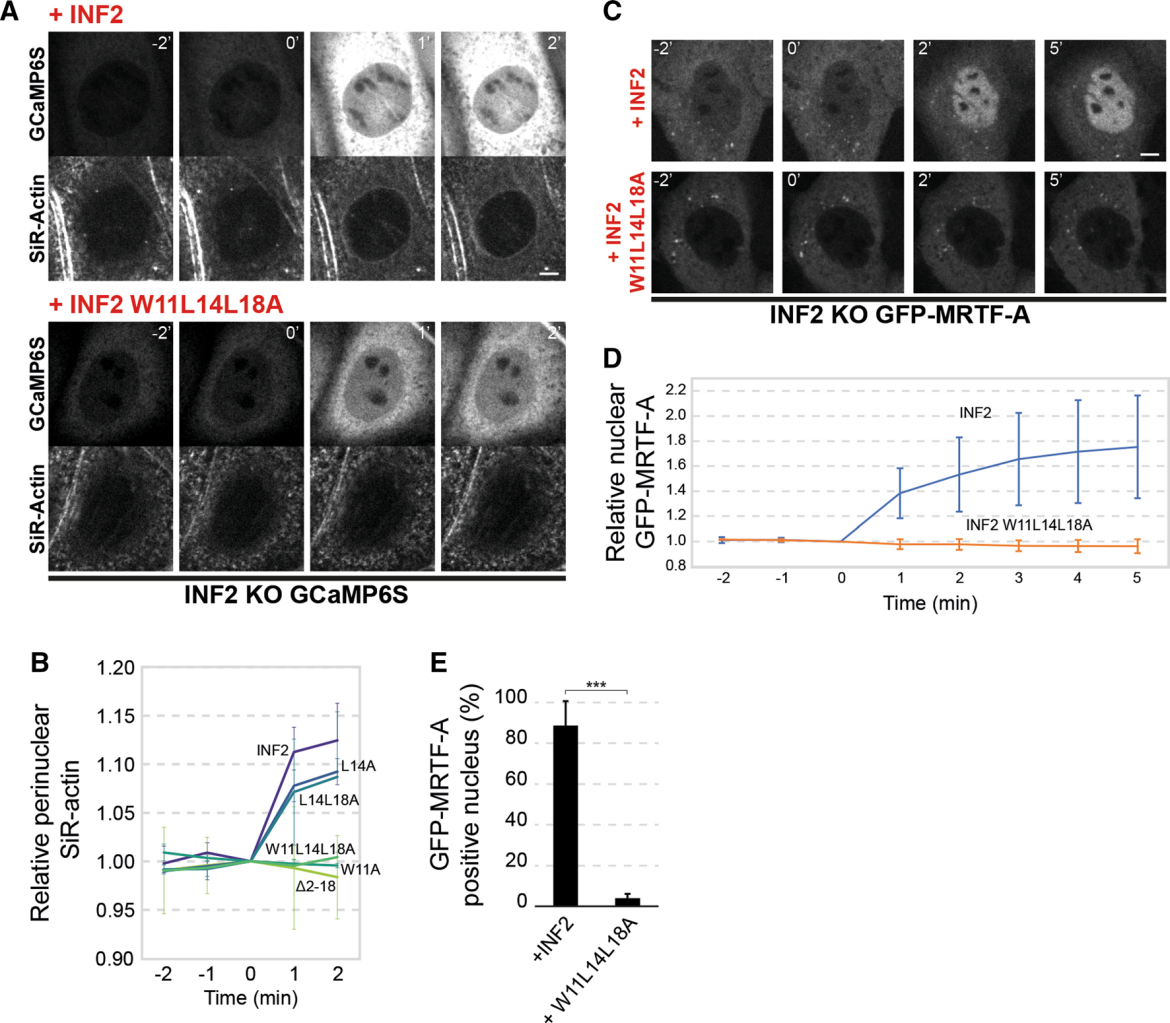
The CaMBS of INF2 is required for actin polymerization and incorporation of GFP-MRTF-A into the nucleus in response to increased intracellular Ca^2+^ levels. **A**, **B** GCaMP6S INF2 KO cells expressing the indicated exogenous INF2 proteins were incubated with SiR-actin to visualize F-actin. Cells were treated with A23187 (0 min) and analyzed by videomicroscopy before and after treatment (**A**). Note that, although the staining of F-actin with SiR-Actin in live cells was poor compared to that of fluorescent phalloidin in fixed cells, SiR-Actin was useful for detecting the initial increase of the levels of F-actin after A23187 stimulation. B Kinetics of the perinuclear content of F-actin as measured by SiR-actin fluorescence relative to that of cells at *t* = 0 min (> 30 cells were analyzed for each experimental condition; three independent experiments). **C**–**E** GFP-MRTF-A INF2 KO cells expressing mCherry fusions of intact INF2 or the INF2 W11L14L18A mutant were treated with A23187 and analyzed by videomicroscopy before and after treatment (**C**). (**D**) Kinetics of the number of INF2 KO cells with nuclear distribution of GFP-MRTF-A relative to that of cells at *t* = 0 min (≥ 30 cells were analyzed for each experimental condition; three independent experiments). Scale bars, 5 μm. (E) The histogram shows the percentage of cells with GFP-MRTF-A predominantly in the nucleus after 5 min of treatment (> 190 cells were analyzed for each experimental condition; 3 independent experiments; ***p < 0.001). The mean ± SD is shown in (** B**, **D**, **E**)

Once the CaMBS was identified, we analyzed how it modulates the functionality of INF2. Consistent with the relative importance of the W11, L14 and L18 residues in the 1-4-8 motif for CaM binding, the expression of intact INF2 efficiently restored the perinuclear F-actin ring and the cytosolic content of F-actin in INF2 KO cells, whereas the efficacy decreased gradually with INF2 L14A and INF2 L14L18A and was negligible with INF2 W11A and INF2 W11L14L18A (Fig. 7A–C). To analyze the effect of the inactivation of the CaMBS in the INF2 response to increased intracellular Ca^2+^ levels, we generated MDCK cells that stably expressed the fluorescent Ca^2+^ sensor GCaMP6S [28]. F-actin was stained in live cells with SiR-Actin to monitor the actin response of the cells to treatment with the calcium ionophore A23187. Since SiR-Actin is a fluorescent derivative of jasplakinolide that, at the concentrations used, might affect actin dynamics and stability [29], we only analyzed the initial burst of actin polymerization. Control and INF2 KO cells had similar levels of the sensor (Fig. S5A) and increased the fluorescent signal of the sensor upon treatment with A23187 (Fig. S5B). Consistent with previous results in other cell lines [16–18, 30], INF2 MDCK KO cells failed to increase actin polymerization upon A23187 treatment, whereas control cells responded rapidly (Fig. S5B, C and Video 1). The expression of INF2 W11L14L18A failed to rescue the defect in actin polymerization, whereas intact INF2 succeeded (Fig. 8A, B, and Video 1). As a control, we checked that INF2 W11L14L18A was not underexpressed relative to exogenous INF2-1 (Fig. S5D, E). The INF2 W11A and INF2 Δ18 mutants behaved similar to W11L14L18A, whereas the effect of INF2 L14A and INF2 L14L18A were similar to that of intact INF2, although they were less efficient (Fig. 8B). These results are consistent with the relative importance of the W11 and the two L residues of the 1-4-8 INF2 motif in the affinity of the 2-19 INF2 peptide for CaM (Fig. 4A–D).

The myocardin-related transcription factor (MRTF) forms a complex with the serum response factor (SRF), a widely expressed transcription factor of mammals, and regulates its activity [31, 32]. At high G-actin concentrations in the cytosol, MRTF forms a reversible complex with G-actin and is held in an inactive state. At low G-actin concentrations, G-actin-free MRTF enters the nucleus and associates with SRF to direct gene transcription [33]. We previously reported that INF2 controls MRTF distribution, and consequently MRTF/SRF-mediated transcription, by modulating the cytosolic levels of free G-actin in human epithelial RPE-1 cells [34]. In HeLa cells, MRTF-A translocates to the nucleus in response to increased Ca^2+^ levels in an INF2 expression-dependent manner [17]. To compare the response of MRTF-A to increased intracellular Ca^2+^ levels in MDCK cells, we transiently expressed GFP-MRTF-A in control and INF2 KO cells and treated them with A23187 for 5 min. Unlike control cells that had GFP-MRTF-A predominantly in the nucleus, MRTF was mostly in the cytoplasm of INF2 KO cells (Fig. S5F, G). Then we used INF2 KO cells stably expressing GFP-MRTF-A to carry out rescue experiments and observed that intact INF2, but not INF2 W11L14L18A, allowed rapid incorporation of GFP-MRTF-A into the nucleus in response to A23187 treatment (Fig. 8C–E and Video 2). In conclusion, the results in Figs. 7 and 8 show that the integrity of the CaMBS of INF2 is necessary for INF2 function at steady state and in response to Ca^2+^ signaling.

### Functional relevance of the calmodulin-binding site of INF2

**Fig. 9:**
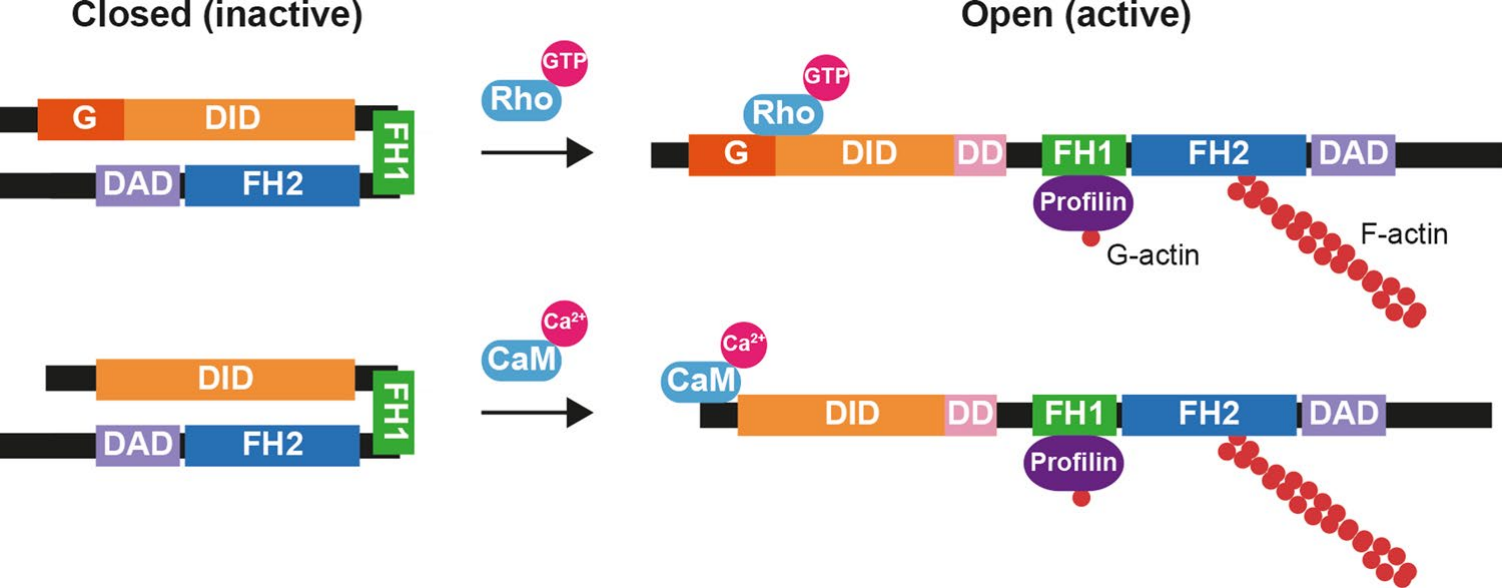
Comparative schematics of the activation of mDia1 and INF2. mDia1 is activated by the binding of GTP-loaded Rho to the GBD, whereas INF2 activation takes place when Ca^2+^/CaM binds to the INF2 N-terminal extension

In INF2, the direct DID–DAD interaction is weak [35], but is considerably strengthened by a complex of lysine-acetylated actin (KAc) and cyclase-associated protein (CAP) [36]. The in vitro interaction of CAP with the DID and of KAc with the DAD led to an inhibition model being proposed in which the complex serves as a bridge between the DID and the DAD [37]. The observation that increased intracellular Ca^2+^ levels produces a transient decrease in lysine acetylation of actin, led to the proposal that Ca^2+^ activates INF2 activation by decreasing the content of KAc and, thereby, reducing the levels of the CAP-Ac inhibitory complex [36]. mDia1 is activated by the binding of Rho to the GBD (G domain and DID) at the N-terminal region of the molecule (Fig. 9, top panel). Due to the slightly overlapping sites of Rho and DAD binding in mDia1, a two-step binding mechanism was postulated in which Rho makes preliminary contact with the G domain forming a loosely bound complex in a first step and, in a second step, interaction with the DID and steric interference and/or charge-charge repulsion lead to dissociation of the DAD [4, 7]. In our analysis of the 2–340 INF2 fragment, we only detected the CaMBS present in the first α-helix of the N-terminal extension. Therefore, it could be that the binding of CaM to this site is sufficient to promote a conformational change in the DID that releases the inhibitory interaction with the DAD mediated by the CAP-KAc complex (Fig. 9, bottom panel). However, our experiments cannot rule out the possibly existence of cryptic CaMBS sites in the region examined that could be unmasked by the binding of CaM to the CaMBS in the first α-helix. Although speculative at this stage, it is also plausible that, by analogy with the two-step mechanism proposed mDia1 activation [4, 7], the binding of the C-terminal lobe of CaM to the CaMBS present in the N-terminal extension of INF2 exposes a cryptic CaMBS in the DID that binds CaM, perhaps through the free N-terminal lobe of the CaM bound to the first α-helix. The binding of CaM to this site would disrupt the DID–CAP interaction, breaking the inhibitory bridge between the DID and the DAD formed by the CAP-KAc complex. Whichever mechanism operates, it would work in concert with decreased KAc levels to rapidly activate INF2 in response to the increased intracellular Ca^2+^concentration [36].

Ca^2+^ is a central second messenger of signal transduction pathways in eukaryotic cells. CaM, which is ubiquitously expressed, acts as a signaling molecule translating transient fluctuations of Ca^2+^ levels inside cells into rapid and appropriate responses [38, 39]. CaM normally constitutes > 0.1% of the total protein present in cells (0.1–1.0 μM) [40], and so does not limit the interaction with INF2. The concentration of intracellular free Ca^2+^ (approximately 0.1 μM) of most cells and the affinity of CaM for Ca^2+^ (*K*_D_ = 0.5–5.0 μM) [40, 41] imply that only a small fraction of CaM is in the form of Ca^2+^/CaM at steady state. According to our results, this pool would be the responsible for the basal activation of INF2 required to maintain the perinuclear F-actin ring and the cytosolic F-actin content that we observe under our cell culture conditions (Figs. 2 and S2A–C). Considering the affinity of the interaction of CaM with the CaMBS-containing INF2 peptide, only a substantial increase in intracellular Ca^2+^ levels—for example that induced by the application of mechanical force or treatment with physiological ligands (e.g., thrombin, lysophosphatidic acid) for G protein-coupled receptors or with calcium ionophore—would be able to mediate Ca^2+^/CaM binding to INF2 for rapid remodeling of the F-actin. This is consistent with previous observations [17, 18, 24, 36] and with our results (Fig. 8A, B).

In summary, we have determined the structure of the N-terminal extension of INF2 and found it to be organized into two α-helices, the first of which contains a Ca^2+^-dependent CaMBS in which the hydrophobic residues involved in the binding are arranged into a 1-4-8 motif that is conserved in vertebrates and in the human population. The identification of the INF2 CaMBS will allow the use of mutants with this site inactivated to distinguish the events mediated by Ca^2^/CaM involving INF2 activation from those that are INF2 independent. Considering the relevance and ubiquity of Ca^2^/CaM signaling, the remodeling of the cytoskeleton by Ca^2^/CaM-mediated INF2 activation, its reported influence on gene expression, and the conservation of the INF2 CaMBS, it is likely that the INF2 CaMBS modulates multiple signaling pathways and physiological processes in vertebrates [17, 24, 42–45].

## Materials and methods

### Materials

**Table:** 

	SOURCE	IDENTIFIER
Antibodies		
Mouse monoclonal anti-CaM	Millipore	Cat#05–173
Rabbit polyclonal anti-Cherry	Abcam	Cat#ab167453
Mouse monoclonal anti-GADPH (clone 6C5)	Ambion	Cat#AM4300
Mouse monoclonal anti-GFP	Sigma-Aldrich	Cat#11814460001
Rabbit IgG control antibody, unconjugated	Sigma-Aldrich	Cat#I5006
Rabbit polyclonal anti-INF2	Andrés-Delgado et al. Madrid et al. [15, 46]	N/A
Secondary donkey anti-mouse, Alexa Fluor 488 conjugated	Thermo Fisher	Cat#A-21202
Secondary donkey anti-rabbit IgG, HRP conjugated	GE Healthcare	Cat#NA934
Secondary donkey anti-mouse IgG, HRP conjugated	Jackson ImmunoResearch Labs	Cat#715–035-151
Bacterial strains		
*E. coli* BL21	Stratagene	Cat#200131
*E. coli* XL10-Gold^a^	Stratagene	Cat#200314
*E. coli* Rosetta 2(DE3)^b^	Merck	Cat#71397
Chemicals, peptides, and proteins		
A23187	Sigma-Aldrich	Cat#C7522
Alexa Fluor™ 488 Phalloidin	Thermo Fisher	Cat#A12379
Calmodulin from bovine brain	Sigma-Aldrich	Cat#208694
Calmodulin from bovine testes	Sigma-Aldrich	Cat#P1431
DAPI	Merck	Cat#268298
Fetal bovine serum	Gibco	Cat#A4766801
Formalin solution, neutral buffered, 10%	Sigma-Aldrich	Cat#HT501128
Fluoromount	Merck	Cat#F4680
G418 sulfate	ChemCruz	Cat#108321–42-2
Glutathione Sepharose^®^ 4B	Cytiva	Cat#GE17-0756–01
Polybrene	Sigma-Aldrich	Cat#TR-1003-G
Polyethylenimine	Polysciences	Cat#23966
Protease inhibitor cocktail	Merck	Cat#11697498001
P rotein A-Sepharose^®^ CL-4B	Cytiva	Cat#GE17-0780–01
Sample buffer, Laemmli 2 × concentrate	Sigma-Aldrich	Cat#S3401
SiR-Actin	Spirochrome	Cat#SC001
Surfactant P20	Cytiva	Cat#BR-1000–54
Triton X-100	Merck	Cat#9036–19-5
T ween-20	Sigma-Aldrich	Cat#9005–64-5
Peptide INF2 2-19^c^ (KGGSVKEGAQRKWAALKEKLG)	Proteomics Facility (CNB, CSIC)	N/A
Peptide INF2 2-19 W11A^c^ KGGSVKEGAQRKAAALKEKLG	Proteomics Facility (CNB, CSIC)	N/A
Peptide INF2 2-19 L14L18A^c^ KGGSVKEGAQRKWAAAKEKAG	Proteomics Facility (CNB, CSIC)	N/A
Peptide INF2 2-19 W11L14L18A^c^ KGGSVKEGAQRKAAAAKEKAG	Proteomics Facility (CNB, CSIC)	N/A
Peptide INF2 2-36^c^ KGGSVKEGAQRKWAALKEKLGPQDSDPTEANLESADPE	pepMic Co., Ltd	N/A
Peptide INF2 2-36^d^ SVKEGAQRKWAALKEKLGPQDSDPTEANLESADPE	CASLO ApS	N/A
Peptide INF2 2-19^d^ SVKEGAQRKWAALKEKLG	CASLO ApS	N/A
Commercial assays		
Lipofectamine 2000	Thermo Scientific	Cat#11668019
QuikChange II Site-Directed Mutagenesis Kit	Stratagene	Cat#200523
Wizard^®^ Plus SV Minipreps DNA Purification Systems	Promega	Cat#A1330
Deposited data		
Chemical shifts of INF2 2-19 in 30% trifluoroethanol	This paper	BMRB-51388
Chemical shifts of INF2 2-19 in aqueous solution	This paper	BMRB-51407
Chemical shifts of INF2 2-19 in 30% trifluoroethanol	This paper	BMRB-51408
Chemical shifts of CaM in aqueous solution	This paper	BMRB-51422
Chemical shifts of INF2 2-19 in 30% trifluoroethanol	This paper	BMRB-51388
Cell lines		
MDCK II	ATCC	Cat#CRL-2936
HEK293T	ATCC	Cat#CRL-3216
Recombinant DNA		
pEGFP-hCaM	Addgene	Cat#47602
pCaM12(D20A/D56A)/pIRES2-eGFP	Addgene	Cat#111512
pCaM34(D93A/D129A)/pIRES2-eGFP	Addgene	Cat#111517
pCaM1234(D20A/D56AD93A/D129A)/pIRES2-eGFP	Addgene	Cat#111518
pEGFP-C1	Clontech	Cat#6084–1
pEGFP-CaM12	This paper	N/A
pEGFP-CaM34	This paper	N/A
pEGFP-CaM1234	This paper	N/A
pGP-CMV-GCaMP6S	Addgene	Cat#40753
pCMS28Cherry	Agromayor et al. [47]	N/A
p33Cherry	This paper	N/A
p3xFLAG-MKL1	Addgene	Cat#11978
pEGFP-MRTF-A	This paper	N/A
pEGFP INF2-1	Andrés-Delgado et al. [46]	N/A
pEGFP INF2-2	Andrés-Delgado et al. [46]	N/A
p33Cherry INF2-1	This paper	N/A
p33Cherry INF2-2	This paper	N/A
p33Cherry INF2-1 Δ2-18	This paper	N/A
p33Cherry INF2-1 Δ2-30	This paper	N/A
p33Cherry INF2-1 W11A	This paper	N/A
p33Cherry INF2-1 L14A	This paper	N/A
p33Cherry INF2-1 L14L18A	This paper	N/A
p33Cherry INF2-1 W11L14L18A	This paper	N/A
MLV-GagPol/pHIV 8.1	Agromayor et al. [47]	N/A
pHIT VSVg	Agromayor et al. [47]	N/A
pGEX-4 T-1	Cytiva	Cat#GE28-9545–49
pGEX-4T-1 INF2 2-340	Andrés-Delgado et al. [46]	N/A
pGEX-4EX	This paper	N/A
pGEX-4EX INF2 30-340	This paper	N/A
pGEX-4EX INF2 19-340	This paper	N/A
pGEX-4EX INF2 19-34	This paper	N/A
pGEX-4EX INF2 2-21	This paper	N/A
pGEX-4EX INF2 4-19	This paper	N/A
pGEX-4EX INF2 6-19	This paper	N/A
pGEX-4EX INF2 8-19	This paper	N/A
pGEX-4EX INF2 4-19 G6R	This paper	N/A
pGEX-4EX INF2 4-19 A7R	This paper	N/A
pGEX-4EX INF2 4-19 Q8A	This paper	N/A
pGEX-4EX INF2 4-19 R9A	This paper	N/A
pGEX-4EX INF2 4-19 K10A	This paper	N/A
pGEX-4EX INF2 4-19 W11A	This paper	N/A
pGEX-4EX INF2 4-19 W11F	This paper	N/A
pGEX-4EX INF2 4-19 W11L	This paper	N/A
pGEX-4EX INF2 4-19 W11V	This paper	N/A
pGEX-4EX INF2 4-19 W11I	This paper	N/A
pGEX-4EX INF2 4-19 A12E	This paper	N/A
pGEX-4EX INF2 4-19 A12Q	This paper	N/A
pGEX-4EX INF2 4-19 A13E	This paper	N/A
pGEX-4EX INF2 4-19 A13Q	This paper	N/A
pGEX-4EX INF2 4-19 A13T	This paper	N/A
pGEX-4EX INF2 4-19 L14A	This paper	N/A
pGEX-4EX INF2 4-19 L14W	This paper	N/A
pGEX-4EX INF2 4-19 L14F	This paper	N/A
pGEX-4EX INF2 4-19 L14V	This paper	N/A
pGEX-4EX INF2 4-19 L14I	This paper	N/A
pGEX-4EX INF2 4-19 K15A	This paper	N/A
pGEX-4EX INF2 4-19 E16A	This paper	N/A
pGEX-4EX INF2 4-19 K17A	This paper	N/A
pGEX-4EX INF2 4-19 L18A	This paper	N/A
pGEX-4EX INF2 4-19 L18W	This paper	N/A
pGEX-4EX INF2 4-19 L18F	This paper	N/A
pGEX-4EX INF2 4-19 L18V	This paper	N/A
pGEX-4EX INF2 4-19 L18I	This paper	N/A
pGEX-4EX INF2 4-19 G19A	This paper	N/A
R701-X65-527: His6-tev-Hs.CALM1(2-148)	Addgene	Cat#159693
Software		
Adobe Illustrator	Adobe	https://www.adobe.com/es/
BIAevaluation Software	Biacore AB	https://biaevaluation.software.informer.com/
Fiji	Open Source	https://fiji.sc/
gnomAD	Broad Institute	https://gnomad.broadinstitute.org/
Microsoft Excel	Microsoft	https://www.microsoft.com/en-us/
NCBI SNP	NCBI	https://www.ncbi.nlm.nih.gov/snp/
NHLBI EVS	NIH NHLBI	https://evs.gs.washington.edu/EVS/
R Studio	Open Source	https://www.rstudio.com/
TOPMed	NIH NHLBI	https://bravo.sph.umich.edu/freeze8/hg38
Topspin	Bruker	https://www.bruker.com/en/products-and-solutions/mr/nmr-software/topspin.html
NMRPipe	Delaglio et al. [48]	https://www.ibbr.umd.edu/nmrpipe/
MddNMR	Orekhov et al. [49]	http://mddnmr.spektrino.com/
NMRFAM-Sparky	UCSF/ Univ. Madison-Wisconsin	https://nmrfam.wisc.edu/nmrfam-sparky-distribution/
Talos-N	NIH NIDDK	https://spin.niddk.nih.gov/bax/nmrserver/talosn/
Cyana 3.98	P. Guntert	http://www.bpc.uni-frankfurt.de/guentert/wiki/index.php/Software
Molmol	Koradi et al. [50]	https://sourceforge.net/p/molmol/wiki/Home/
Haddock 2.4	Univ. Utrecht	https://wenmr.science.uu.nl/haddock2.4/
Other		
Sensor Chip SA	Cytiva	BR100032
µ-Slide 8 Well	Ibidi	Cat#80826

^a^For expression of GST fusion proteins

^b^For expression of recombinant CaM for NMR analysis spectroscopy

^c^For surface plasmon resonance analysis

^d^For NMR spectroscopy

### Cells and cell culture

Epithelial canine MDCK II cells, MDCK II INF2 KO cells [20] and human epithelial HEK293T cells were grown in MEM supplemented with 5% or 10% (v/v), respectively, fetal bovine serum (FBS) at 37 °C in a 95% air/5% CO_2_ atmosphere. Mycoplasma testing was regularly performed.

### Molecular cloning

The polylinker of the GST vector pGEX-4T1 was modified by insertion of the polylinker sequence 5´-GAATTCTCCGGAGGCGGCCGCTCTAGACTCGAG-3´, using annealed oligonucleotides, generating the pGEX-4EX plasmid. The inserted sequence contains one BspEI site and one XhoI site at the 5´ and 3´ end, respectively, that were used for cloning. For expression in *E. coli* as GST fusions, the INF2 DNA fragments 18–340 and 30–340 were obtained by PCR using the appropriate forward and reverse oligonucleotide primers with a BspEI site (forward primer) and a XhoI site (reverse primer) at their 5´ end, and INF2-1 cDNA as the template. The products were cloned between the BspEI and XhoI sites of pGEX-4EX. For the human INF2 2–21, 4–19, 6–19 and 8–19 GST constructs and derivative mutants, the appropriate forward and reverse oligonucleotide with preformed 5´ BspEI (forward oligonucleotide) and XhoI (reverse oligonucleotide) cohesive ends were annealed and cloned between the BspEI and XhoI sites of pGEX-4EX. For the pull-down experiments with GST-CaM, we generated a DNA construct by cloning the XhoI/BamHI fragment, which contains the CaM coding sequence, from pEGFP-hCaM (Addgene #47602) in pGEX-4T1.

For mammalian expression, the polylinker of the retroviral pCMS28Cherry vector [47] was modified by deletion of the XhoI–BamHI fragment, which contains an internal ribosomal entry site and the puromycin resistance gene, elimination of the unique BamHI and SalI sites, and insertion of the polylinker sequence used to generate pGEX-4EX, to give rise to the p33Cherry vector. For expression as mCherry fusions, the entire coding sequence of human INF2-1 and INF2-2 obtained from pEGFP-C1 INF2-1 and pEGFP-C1 INF2-2 was excised with BspEI and XhoI and cloned in-frame of the mCherry coding sequence from p33Cherry. The p33Cherry INF2-1 Δ2–18 and Δ2–30 constructs were obtained using the BspEI–SalI DNA fragment from pGEX-4EX INF2 18–340 and 30–240 constructs, respectively, to replace the corresponding BspEI–SalI fragment of p33Cherry INF2-1, using the unique SalI site present in the sequence encoding the DID of human INF2. For the W11A, L14A, L14L18A and W11L14L18A INF2 mutants in p33Cherry the INF2 fragment used was generated by digestion with BspEI and SalI of PCR products, obtained using forward and reverse primers with the appropriate modifications, and INF2-1 as template.

The constructs pEGFP-CaM12, 34 and 1234 were obtained by replacing the XhoI–BamHI CaM insert in pEGFP-hCaM with the corresponding XhoI–BamHI CaM DNA fragment obtained from pCaM12(D20A/D56A)/pIRES2-eGFP, pCaM34(D93A/D129A)/pIRES2-eGFP and pCaM1234(D20A/D56AD93A/D129A)/pIRES2-eGFP [51]. pGFP-MRTF-A was obtained by in-frame cloning the MRTF-A EcoRI–BamHI insert from p3xFLAG-MKL1 into pEGFP-C1. All constructs were verified by DNA sequencing (Macrogen).

### Retroviral infections

To pack retroviral plasmids into retroviral particles, HEK293T cells in a p100 culture dish were cotransfected with p33Cherry constructs expressing the indicated mCherry-INF2 fusions and the plasmids MLV-GagPol/pHIV 8.1 and pHIT VSVg (1.4 μg/ml) in a 4.6:3.3:1 ratio using polyethyleneimine [47]. After 48 h the supernatant containing the viral particles was collected and filtered. For expression of mCherry alone or mCherry-fused INF2 proteins, MDCK cells were treated with 10 μg/ml polybrene at 37ºC for 15 min and incubated with the supernatant containing the viral particles for 7 h. After that, cells were washed and incubated for 48 h.

### Transfections and generation of stable transfectants

MDCK or MDCK KO cells stably expressing exogenous proteins (GCaMP6S or GFP-MRTF-A) were generated by transfection using Lipofectamine 2000 according to the manufacturer’s instructions. 48 h later, single cells expressing GFP were FACS sorted. In the case of MDCK INF2 KO cells stably expressing GFP-MRTF-A, but not in those expressing GCaMP6S, transfected cells were selected with 1 mg/ml G-418 for 14 days. Individual clones were screened by fluorescence microscopy and immunoblotting.

### Confocal microscopy and videomicroscopy

Cells were fixed with 10% formalin (37% formaldehyde solution) and permeabilized with 0.2% Triton X-100 for 5 min on ice. After blocking with 3% (wt/vol) BSA for 30 min, cells were stained with phalloidin-Alexa 488 and DAPI, and then washed extensively. Coverslips were mounted on glass slides with Fluoromount. Fluorescence was examined with a Nikon A1R + confocal laser-scanning microscope with 60x (NA 1.2) water objective. Brightness and contrast were optimized with Fiji software (National Institutes of Health, USA). LSM images were converted to TIFF format. The levels of perinuclear F-actin were measured using a ROI consisting of a 0.5-μm-width ring around the nucleus, while those of cytosolic F-actin were measured in a 20 μm^2^ square ROI close to the central region of the cell. The data represented correspond to the average intensity of three planes. The images obtained were processed using Fiji (ImageJ). We used videomicroscopy to monitor actin dynamics using SiR-Actin, which is a fluorogenic reagent that allows labeling of F-actin in live cells [29]. SiR-actin emits in far-red because of its silicon rhodamine moiety, thereby making it compatible with the emission of the GFP (GCaMP6S) and Cherry (INF2) proteins expressed in our experiments. SiR-Actin (0.6 μM) was added to cells expressing GCaMP6S 1 h before recording. Verapamil (10 μM), an efflux pump inhibitor, was added simultaneously to increase the otherwise very low staining efficiency of MDCK cells. These cells or cells expressing GCaMP6S or GFP-MRTF-A were plated onto µ-Slide 8 well dishes, and maintained at 37 °C in MEM without phenol red supplemented with 12.5 mM HEPES, pH 7.0, and 5% (v/v) FBS during the recording. To increase the intracellular Ca^2+^ levels, cells were treated with 8 μM A23187 (time = 0). Cells were filmed with an Olympus Spinning Disk SpinSR10 with 60x (NA 1.3) silicone immersion objective. Brightness and contrast were optimized with ImageJ (https://imagej.nih.gov/ij/). Quantifications were performed using ImageJ software.

### GST pull-down assay

For use in pull-down assays, GST-fused proteins were expressed in *E. coli* BL21 cells. Cells were grown in LB media at 37 °C until the culture reached an OD_600_ of 0.6–0.8, whereupon the temperature was decreased to 20 °C and 0.5 mM IPTG was added to induce expression of the recombinant protein for 16 h. Cell lysates of HEK293T cells expressing GFP-CaM or mCherry-INF2 proteins were incubated in assay buffer (PBS supplemented with 1 mM DTT, 0.1 mM PMSF, 0.2% Tween-20, 0.5 mg/ml DNase I, 5 mM Ca^2+^, and a commercial cocktail of protease inhibitors) at 4 ºC with 30 µg of the indicated GST-fused proteins immobilized on glutathione-Sepharose beads. After 3 h, the beads were collected by centrifugation in a microfuge at 800 rpm for 5 min, and washed twice with cold PBS. Samples were analyzed by immunoblotting. An aliquot from each glutathione-Sepharose bead-immobilized GST-fused protein was stained with Coomassie blue to control for the amount of GST protein used.

### Immunoblotting

After blocking with 5% BSA (*w*/*v*) and 0.05% (v/v) Tween-20 in Tris-buffered saline, membranes were incubated overnight with the indicated primary antibodies, washed with Tris-buffered saline containing 0.05% Tween-20, and incubated for 30 min with the corresponding secondary antibodies coupled to HRP. The primary antibody used to detect INF2 recognizes both endogenous canine INF2 and exogenous human mCherry-INF2 [15, 46]. The signal was visualized with Clarity Western ECL substrate (BioRad).

### Immunoprecipitation analysis

HEK293T cells expressing mCherry fusions of INF2 or INF2 W11L14L18A mutant were lysed at 4 ºC in 1 ml lysis buffer containing 20 mM Tris, pH 8.0, 150 mM NaCl, 5 mM CaCl_2_, 1.15% (v/v) glycerol, 1% (v/v) Nonidet P-40, 1 mM sodium orthovanadate, 0.1 mM phenylmethylsulphonyl fluoride, and a commercial cocktail of protease inhibitors. The cell extracts centrifuged in a microfuge at 14,000 rpm for 5 min, and the cleared supernatant was then incubated with control or anti-Cherry rabbit antibodies bound to protein A-Sepharose beads for 3 h. After four washing steps, bound proteins were eluted in Laemmli’s buffer and processed for immunoblotting to detect exogenous INF2 with anti-Cherry antibodies and endogenous CaM with anti-CaM antibodies.

### Synthetic peptides

For use in surface plasmon resonance analysis, the peptide corresponding to amino acids 2-19 of human INF2 and similar peptides with replacements in the W11, L14 and L18 residues were synthesized in an automated multiple peptide synthesizer (Multipep, Intavis, Köln, Germany) in the Proteomics Facility of the Centro Nacional de Biotecnología (CSIC, Madrid). The INF2 2–36 peptide was obtained as a bespoke product with the same modifications from pepMic (Suzhou, China). These peptides contained two G residues at its N terminus to separate the INF2 sequence from an additional N-terminal end K residue, which was biotinylated in the ε-amino group. For use in NMR studies, the INF2 2–36 and 2-19 peptides were synthesized to order, without any modification, by CASLO ApS (Denmark). All the peptides were synthesized using the solid-phase synthetic procedures and standard Fmoc [*N*-(9-fluorenyl)methoxycarbonyl] chemistry [52], and then underwent reverse-phase HPLC purification.

### Surface plasmon resonance analysis

All surface plasmon resonance experiments were carried out at 25ºC in a Biacore 3000 (Cytiva) robotic biosensor. Analyses were performed using HBSP (10 mM HEPES pH 7.4, 150 mM NaCl, 0.005% surfactant P20) supplemented with 1 mM CaCl_2_ as running buffer and for the dilution of analytes and ligands. Biotinylated peptides were individually immobilized on a streptavidin-coated sensor chip in flow cells 2, 3 and 4 at a flow rate of 10 µl/min (20–50 RU captured). The empty flow cell 1 was used as a control for non-specific binding and bulk effects. Purified CaM was injected at different concentrations ranging from 13.2 nM to 9.6 µM in duplicate, at a flowrate of 50 µl/min, with an association time of 60 s followed by dissociation for 60 s. After that, the sensor chip was regenerated by injection of 10 mM glycine–HCl, pH 1.7, with a contact time of 12 s. The signal obtained in the reference cell was subtracted from each of the sample sensorgrams and, afterwards, the signal corresponding to the injection of the buffer was also subtracted. Sensorgrams with different concentrations of CaM were overlaid, aligned and analyzed with BIAevaluation Software 4.1, using the double-reference method [53]. In all sensorgrams, the binding reached the equilibrium very quickly and the complete dissociation was achieved in a few seconds, due to the very fast association (K_a)_ and dissociation (*K*_d_) constant rates, which were beyond the detection limits of the equipment. Therefore, we used the response signal at equilibrium at each concentration of CaM to calculate the affinity constant (*K*_D_). A Langmuir binding isotherm was fitted to determine *K*_D_ using the equation: *R*_eq_ = *C* × *R*_max_/*C* + *K*_D,_ where *C* is the analyte concentration and *R*_max_ is the maximal binding capacity.

### Expression and purification of CaM

For NMR spectroscopy, recombinant human CaM was produced using the plasmid R701-X65-527: His6-tev-Hs.CALM1(2–148) [54] expressed in *E. coli* Rosetta 2(DE3) cells. Isotopically labeled CaM was produced in supplemented minimal growth medium containing ^15^NH_4_Cl and/or ^13^C-glucose as the sole sources of nitrogen and carbon respectively, as previously described [55]. Briefly cells were grown in 2 L of LB media at 37 °C until and OD_600_ of 0.8 was obtained, whereupon they were centrifuged at 3000×*g* for 20 min and the pellet was resuspended in 0.5 ml of prewarmed, isotopically labeled minimal medium. Cells were incubated at 37 °C for 1–2 h, and the temperature was then decreased to 20 °C before induction with 0.5 mM IPTG. The cells were harvested after 16 h. For CaM purification, the pellet obtained by centrifugation was lysed by mechanical disruption through sonication and the supernatant was loaded into a nickel-based HisTrap FF Crude column (GE Healthcare) using buffer A (50 mM Tris–HCl pH 8.0, 300 mM NaCl and 10 mM imidazole). Protein was eluted with an imidazole gradient in buffer A from 10 mM up to 250 mM. Fractions containing CaM were dialyzed against buffer B (20 mM Tris–HCl pH 8.0, 10 mM NaCl) and digested with TEV-protease produced in-house using the protocol of Blommel and Fox [56]. The extent of the digestion was confirmed by the difference in the size of noncleaved and cleaved CaM, as assessed by SDS-PAGE. After removing the remaining uncleaved protein in a HisTrap FF Crude column, the cleaved product was buffer exchanged using a Hi-Prep desalting column (GE Healthcare) into the final buffer (20 mM Tris–HCl pH 8.0, 10 mM NaCl and 5 mM CaCl_2_ pH 7.0). Eluted fractions containing CaM were concentrated to an appropriate volume and the sample concentration was estimated using a Nanodrop spectrophotometer. The identity of CaM was confirmed by MALDI-TOF mass spectrometry.

### NMR spectroscopy of peptides

The deuterated compounds [D_3_]-2,2,2-trifluoroethanol (TFE) (99%) and D_2_O (99.9%) were purchased from Eurisotop, France*.* Peptide samples for NMR spectra acquisition were prepared at 1.0 mM concentration in a final volume of 500 μl of either H_2_O/D_2_O (9:1 v/v) or 30% [D3]-TFE/70% H_2_O/D_2_O (9:1 v/v). The pH was measured with a glass microelectrode and adjusted to the desired value of 5.5 by adding NaOD or DCl and was not corrected for isotope effects. All samples contained 1.5 µl of sodium 2,2-dimethyl-2-silapentane-5-sulfonate (DSS) as an internal reference for ^1^H chemical shifts. Samples were placed in 5-mm NMR tubes. ^1^H 1D and 2D spectra of peptides were recorded on a Bruker AVNEO-600 spectrometer operating at a proton frequency of 600.13 MHz and equipped with a cryoprobe. The acquired 2D spectra were: ^1^H,^1^H double-quantum filtered correlation spectroscopy (COSY), ^1^H,^1^H total correlation spectroscopy (TOCSY), ^1^H,^1^H nuclear Overhauser effect spectroscopy (NOESY) and ^1^H–^13^C heteronuclear single quantum coherence (HSQC) spectra at ^13^C natural abundance. The water signal was suppressed using an excitation sculpting scheme [57]. All spectra were processed using Topspin 4.0.8 program. Baseline correction was applied in both dimensions. ^13^C *δ*-values were indirectly referenced using the IUPAC-IUB recommended ^1^H/^13^C chemical shift ratio (0.25144953) [58]. ^1^H and ^13^C chemical shifts for the peptides were assigned by analyzing the 2D NMR spectra and with the aid of the NMRFAM-SPARKY software [59]. The chemical shift values were deposited in BioMagResBank under accession codes 51,388–51,389 for peptide INF2 2-19 and 51,407–51,408 for peptide INF2 2–36.

### NMR spectroscopy of CaM

Protein samples for NMR spectrum acquisition were prepared by adding 50 μl D_2_O to a 450 μl of protein in the final buffer (20 mM Tris–HCl, pH 7.0; 10 mM NaCl; 5 mM CaCl_2_). Protein concentration was approximately 250 μM. 2D ^1^H,^15^ N-HSQC and series of 3D spectra NHCO, HN(CA)CO, HNCA, HN(CO)CA, HNCACB, and CBCA(CO)NH were acquired using ^13^C,^15^ N-CaM samples on a Bruker AVNEO-800 spectrometer operating at proton frequencies 800.1 MHz, and equipped with a TCI cryoprobe. The acquisition parameters for these spectra are presented in Table S2. All spectra were processed using Topspin 4.0.8 program A methanol sample was employed to calibrate cryoprobe temperature. ^1^H, ^15^ N and ^13^C chemical shifts for backbone atoms and for C_β_ carbons of free CaM and for the CaM/INF2-2-19 complex were assigned by analysis of the series of acquired 3D spectra with the aid of the NMRFAM-SPARKY software [59]. The chemical shift values were deposited in BioMagResBank under accession code 51,422.

### Determination of secondary structure

The elements of the secondary structure of peptides and proteins can be determined from the Δ*δ*_Hα_ and Δ*δ*_Cα_ values, which are the differences in the chemical shifts of ^1^Hα protons and ^13^Cα carbons from reference values characteristic of random coil. Thus, Δ*δ*_Hα_ = *δ*_Hα_^observed^ – *δ*_Hα_^RC^, ppm, and Δ*δ*_Cα_ = *δ*_Cα_^observed^ – *δ*_Cα_^RC^, ppm, where *δ*_Hα_^observed^ and *δ*_Cα_^observed^ are the observed chemical shifts for ^1^Hα and ^13^Cα nuclei, respectively, and *δ*_Hα_^RC^ and *δ*_Cα_^RC^ are reference random coil values for the ^1^Hα and ^13^Cα chemical shifts, respectively. These reference values were taken from Wishart et al. [60]. α-helices are identified by stretches of negative Δ*δ*_Hα_ and positive Δ*δ*_Cα_ values, and extended/β-strand segments by positive Δ*δ*_Hα_ and negative Δ*δ*_Cα_ values. Values within the ranges − 0.04 ppm < Δ*δ*_Hα_ <  + 0.04 ppm and − 0.4 ppm < Δ*δ*_Cα_ <  + 0.4 ppm are considered to indicate random coil. The helix populations can be estimated from the Δ*δ*_Hα_ values averaged for the helical residues—amino acids 3–17 in the case of the N-terminal helix of the INF2 peptides—divided by the Δ*δ*_Hα_ value corresponding to 100% helix (− 0.39 ppm), that is, % helix = 100 × ⟨Δ*δ*_Hα_⟩/(− 0.39) [61]. Thus, the populations of the N-terminal helix formed by both peptides are approximately 23% in aqueous solution, increasing to 43–47% in the presence of TFE. Assuming an experimental error of ± 0.01 ppm in the measurement of ^1^H chemical shifts, the error in the estimated populations would be ± 3%.

### Peptide structure calculation

Peptide structures were calculated using the standard iterative protocol for automatic NOE assignment of the CYANA 3.98 program [62], which performs seven cycles of combined automated NOE assignment and structure calculation, being 100 conformers calculated per cycle. The experimental input data were the lists of: (1) assigned chemical shifts, (2) NOE integrated cross-peaks present in 150 ms NOESY spectra, and (3) *ϕ* and *ψ* dihedral angle restraints. The automatic integration subroutine of SPARKY software (T. D. Goddard and D. G. Kneller, SPARKY 3, University of California, San Francisco) was used to integrate NOE cross-peaks. Restraints for dihedral angles were obtained from ^1^H and ^13^C chemical shifts using the TALOSn webserver [63]. The final structure of each peptide is the ensemble of the 20 lowest target function conformers calculated in the final cycle. Table S1 provides a summary of the statistical parameters of these structural ensembles, which were visualized and examined using the MOLMOL program [50]. Structures are available upon request from the authors.

### Titration of CaM with INF2-2-19 peptide

To determine experimentally how CaM and the N-terminal region of INF2 interact, a ^15^ N-CaM sample was titrated with increasing quantities of INF2-2-19 peptide. 2D [^1^H-^15^ N]-HSQC spectra were acquired at 25 °C with protein/peptide ratios of 1:0, 1:0.1, 1:0.2, 1:0.5, 1:1, 1:2, 1:4 and 1:8. Since most cross-peaks in the [^1^H-^15^ N]-HSQC spectra were shifted upon peptide titration (Fig. S3C), to confirm cross-peak assignment a ^13^C,^15^N-CaM/INF2-2-19 sample at 1:1 ratio was used to acquire a series of 3D spectra (HNCA, HN(CA)CO, HN(CO)CA, CBCANH, CBCA(CO)NH and NHCO), which were acquired using non-uniform sampling (NUS) and were processed using NMRPipe [48] and MddNMR software (http://mddnmr.spektrino.com) [49]. Details of the acquisition parameters of these spectra are presented in Table S2. Analysis of these spectra enabled the almost complete assignment of ^1^H, ^15^ N, and ^13^C backbone resonances of CaM in its complex with the INF2 2-19 peptide. Chemical shift perturbations (CSPs) upon peptide titration were calculated using the equation CSP = [(Δ*δ*_1H_)^2^ + (Δ*δ*_15N_/5)^2^ × 0.5]^1/2^, in which Δ*δ*_1H_ and Δ*δ*_15N_ are the chemical shift changes in the amide ^1^H and ^15^ N resonances, respectively. Using the Schumann criteria [64], the threshold for significant changes was 0.25 ppm.

### NMR-driven docking (HADDOCK)

To visualize how CaM–INF2 interaction occurs, a complex model was built using the Haddock webserver [65, 66]. This program requires input coordinates for both, protein and peptide, and a definition of their interaction residues, classified as active or passive. Protein coordinates were taken from pdb code 3CLN. Peptide coordinates were those of the lowest target function conformer of the structural ensemble calculated in this work. The active residues for the INF2-2-19 peptide were those determined by the pull-down analysis carried out in this work, and those for the protein were the ones exhibiting the greatest chemical shift perturbations (CSP; Δδ^av^ > 0.25 ppm; see Fig. S3D). Representative complexes were those with the best Haddock docking scores. Figure 6A shows the structure with the lowest Haddock docking score.

### Bioinformatic analyses

The protein encoded by the *INF2* gene in the Human Genome Resources at the National Center for Biotechnology Information (NCBI) (https://www.ncbi.nlm.nih.gov/genome/guide/human) was taken as the human INF2 reference for sequence comparisons. NCBI blast was used to align the human INF2 sequence with that of the indicated vertebrates (Fig. 5A). INF2 sequences from more than 300 different vertebrates were analyzed in Fig. S3A. Only the variants found in at least two different species were considered. The following databases were searched to find INF2 variants in the human population: gnomAD, LOVD3, NCBI Clin Var, NCBI SNP, NHLBI EVS and TOPMed (Fig. 5B). The CaM-binding protein database (Gene Ontology, GO:0,005,516) and the CaM target database (http://calcium.uhnres.utoronto.ca/ctdb/ctdb) were interrogated to identify proteins with a Ca^2^/CaM binding motif similar to the INF2 1-4-8 motif.

### Statistical analysis

Most graphs and statistical analysis were performed with RStudio, version 1.2.5033 software. Statistical significance of differences between means was assessed with two-tailed Student’s unpaired *t*-tests, while differences between median values were examined using the Mann–Whitney–Wilcoxon test, as indicated. Additional information is provided in the figure legends.

## Supplementary Information

Below is the link to the electronic supplementary material.

Supplementary file1 (PDF 8208 KB)

Video 1. Effect of the expression of normal INF2 or INF2 proteins with CaMBS mutations on actin dynamics in INF2 KO cells. Control GCaMP6S cells or INF2 KO GCaMP6S cells expressing or not mCherry fusions of normal INF2 or the INF2 W11L14L18A mutant were stained with SiR-Actin and treated with A23187 (0’). The signal corresponding to GCaMP6S and SiR-Actin is shown

Video 2. Effect of the expression of normal INF2 or the W11L14L18A INF2 mutant on MRTF-A distribution. INF2 KO GFP-MRTF-A cells expressing mCherry fusions of normal INF2 or the W11L14L18A INF2 mutant were treated with A23187 (0’). The signal corresponding to GFP-MRTF-A is shown

## Data Availability

The datasets generated during and/or analyzed during the current study are available from the corresponding author upon request.
