# Supplementary material for: Structure and function of the N-terminal extension of the formin INF2
Source: Cell Mol Life Sci. 2022 Oct 28;79(11):571. doi: 10.1007/s00018-022-04581-y (PMC9616786; doi:10.1007/s00018-022-04581-y)
Supplement: Supplementary file 1 — Supplementary file1 (PDF 8208 KB) [file 18_2022_4581_MOESM1_ESM.pdf]

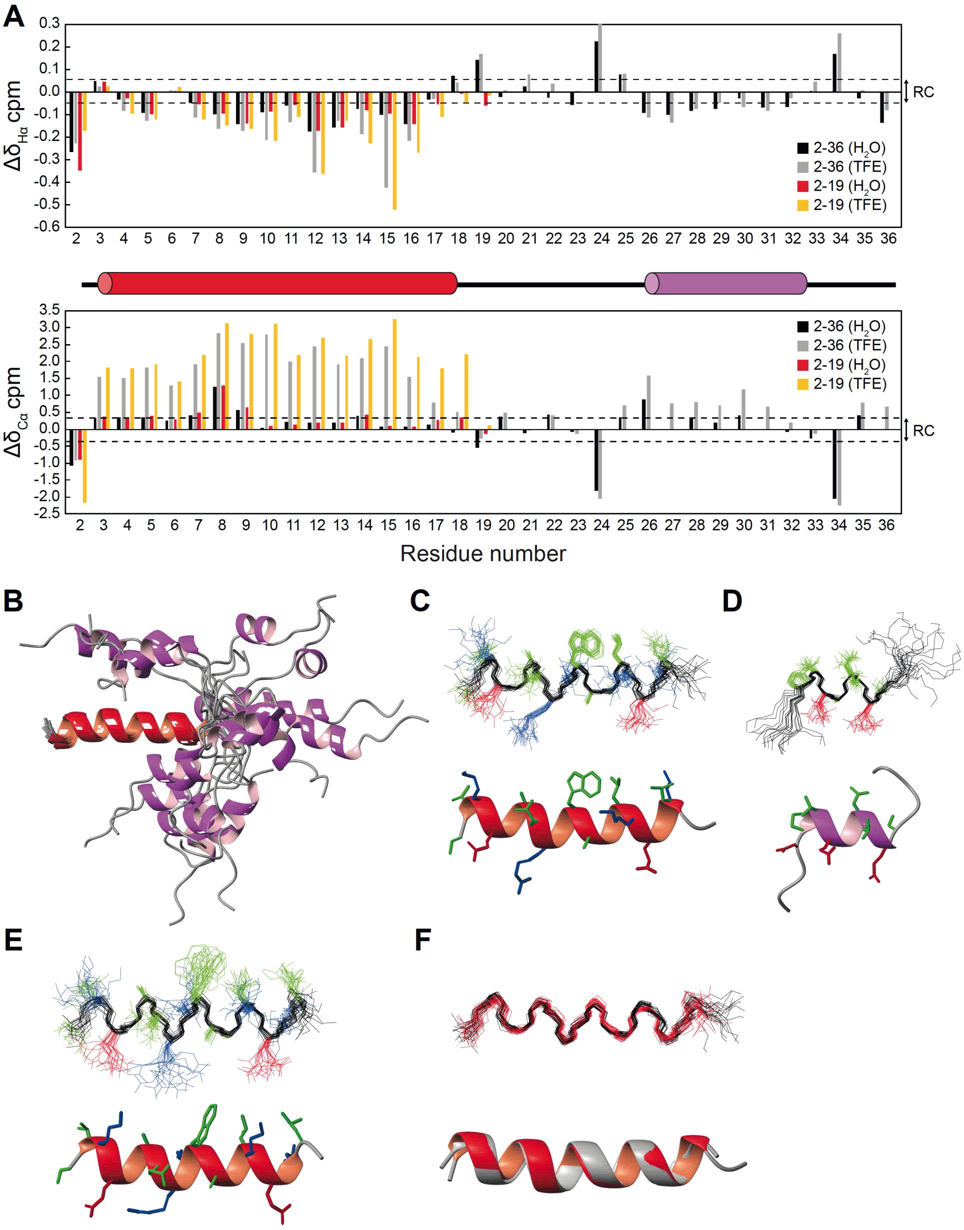

Figure S1. **NMR data and structures of the INF2 2-36 and 2-19 free peptides**. (**A**) Bar plots of the difference of chemical shift values for α ^{1}H ($\Delta\delta_{H\alpha}$) and ^{13}C ($\Delta\delta_{C\alpha}$) as a function of the amino acid sequence of the 2-36 peptide in aqueous solution (black bars) and in 30% TFE (grey bars), and of 2-19 peptide in aqueous solution (red bars) and in 30% TFE (gold bars). The stretches of negative $\Delta\delta_{H\alpha}$ values and positive $\Delta\delta_{C\alpha}$ values indicate helix formation, as schematically shown in the cartoon between the two panels. RC, random coil value interval. (**B**) Ribbon representation of the ensemble of the 20 lowest target function conformers calculated for the INF2 2-36 peptide on the basis of NMR parameters. The conformers are overlaid onto the first helix (in red). The second helix is colored in magenta. (**C**) First helix of INF2 2-36: overlay of backbone atoms for residues 3-17 and ribbon representation of the first conformer of the ensemble. (**D**) Second helix of INF2 2-36: overlay of backbone atoms for residues 25-31 and ribbon representation of the first conformer of the ensemble. (**E**) Helix of INF2 2-19: overlay of backbone atoms and ribbon representation of the first conformer of the ensemble. (**F**) Overlay of the INF2 2-19 helix (red) onto the first helix of INF2 2-36 (black gray). Top panel: Backbone atoms of the 20 lowest target conformers of the two ensembles. Bottom panel: Ribbon representations of the first conformer of each ensemble. In panels (C-E), backbone atoms are shown in black. K and R side chains are displayed in blue, D and E side chains in red, and all others in green.

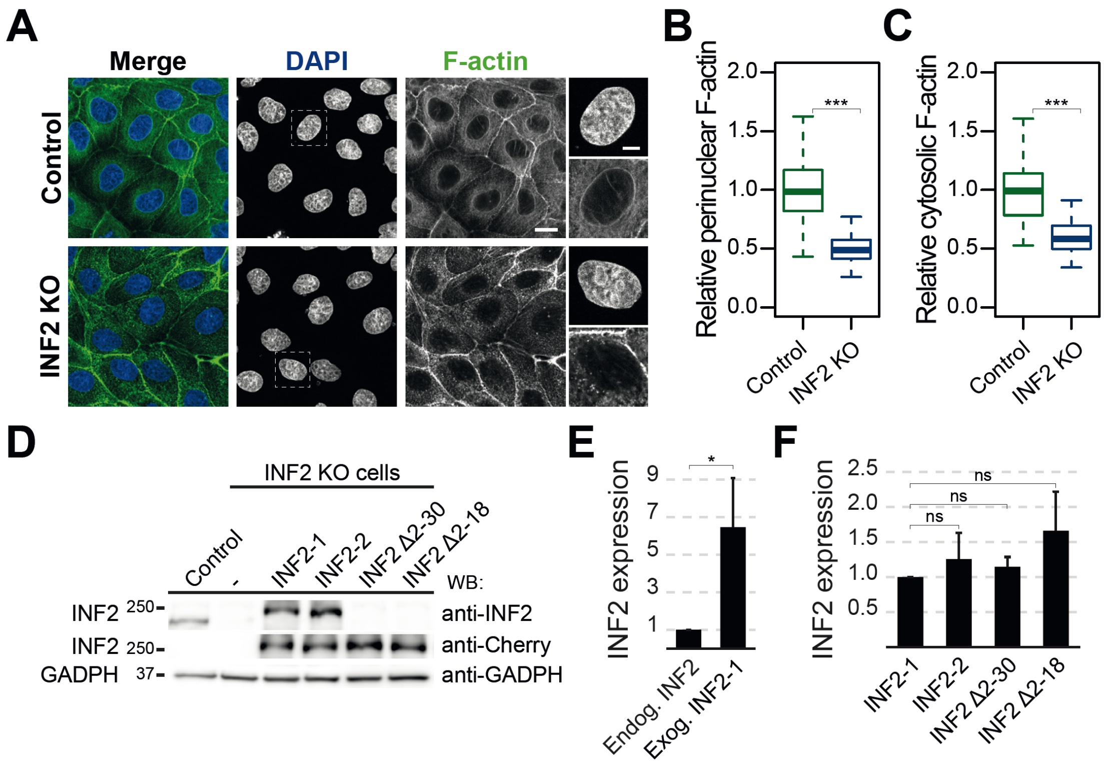

Figure S2. **Distribution of F-actin in INF2 KO cells and analysis of the levels of expression of exogenous INF2**. **(A-C)** Control and INF2 KO cells were stained for F-actin. Nuclei were visualized with DAPI. An enlargement of the DAPI and F-actin staining corresponding to the boxed regions is shown in the rightmost panels. Scale bars, 15 µm for the panoramic views and 5 µm for the enlargements (A). Box plots showing the intensity of F-actin staining at the perinuclear region (B) and the cytosol (C) relative to that of control cells (> 160 cells were examined for each experimental condition; three independent experiments; ***, $p < 0.001$). (**D-F**) The expression of endogenous INF2 in control wild type cells and that of the indicated exogenous INF2 proteins in INF2 KO cells were analyzed by immunoblotting with anti-antibodies that recognize both canine and human INF2 (top panel). The exogenous proteins were also analyzed with anti-Cherry antibodies (middle panel). The levels of GAPDH were taken as a loading control (bottom panel) (D). The graphs show the expression of exogenous INF2-1 relative to that of endogenous INF2 (E), and the expression of exogenous INF2-2, INF2 Δ2-30 and INF2 Δ2-18 relative to that exogenous INF2-1 (F). Three independent experiments; ns, not significant; *, $p < 0.05$).

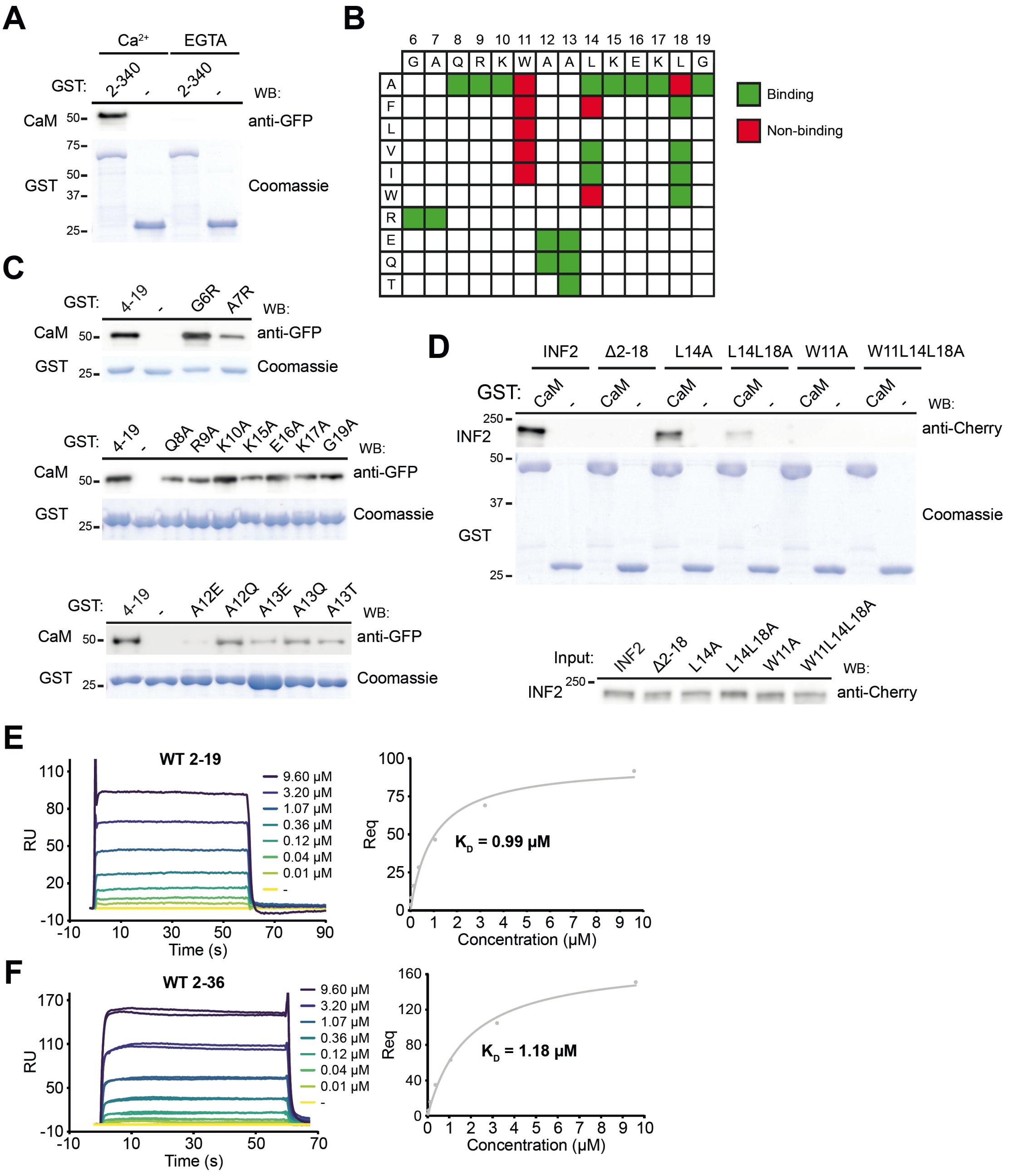

Figure S3. **Mutational analysis of the 2-19 INF2 peptide**. (**A**) Pull-down analysis of the association of GFP-CaM with a GST fusion of the 2-340 fragment of INF2 in the presence of 5 mM Ca^{2+} or 7.5 mM EGTA. (**B**) Summary of the association of GFP-CaM with GST fused to the 4-19 fragment of INF2 with the indicated amino acid replacements. (**C**) Representative pull-down experiments of the association of GFP-CaM with GST fusions of the intact 4-19 fragment of INF2 or with the indicated amino acid replacements. (**D**) A GST fusion of CaM was used in pull-down experiments with full-length INF2 either intact or with the indicated amino acid replacements. The purified GST fusions were stained with Coomassie blue as a control for the amount of GST protein used in (A, C, D). (**E**, **F**) Representative surface plasmon resonance analysis of the interaction of purified CaM from bovine testes (E) or brain (F) with immobilized 2-19 (E) or 2-36 (F) peptides in the presence of Ca^{2+} (left panels). The corresponding equilibrium plots, with indication of the apparent K_D resulting from the same experiment, are shown (right panels). RU, resonance units; Req, RU at equilibrium.

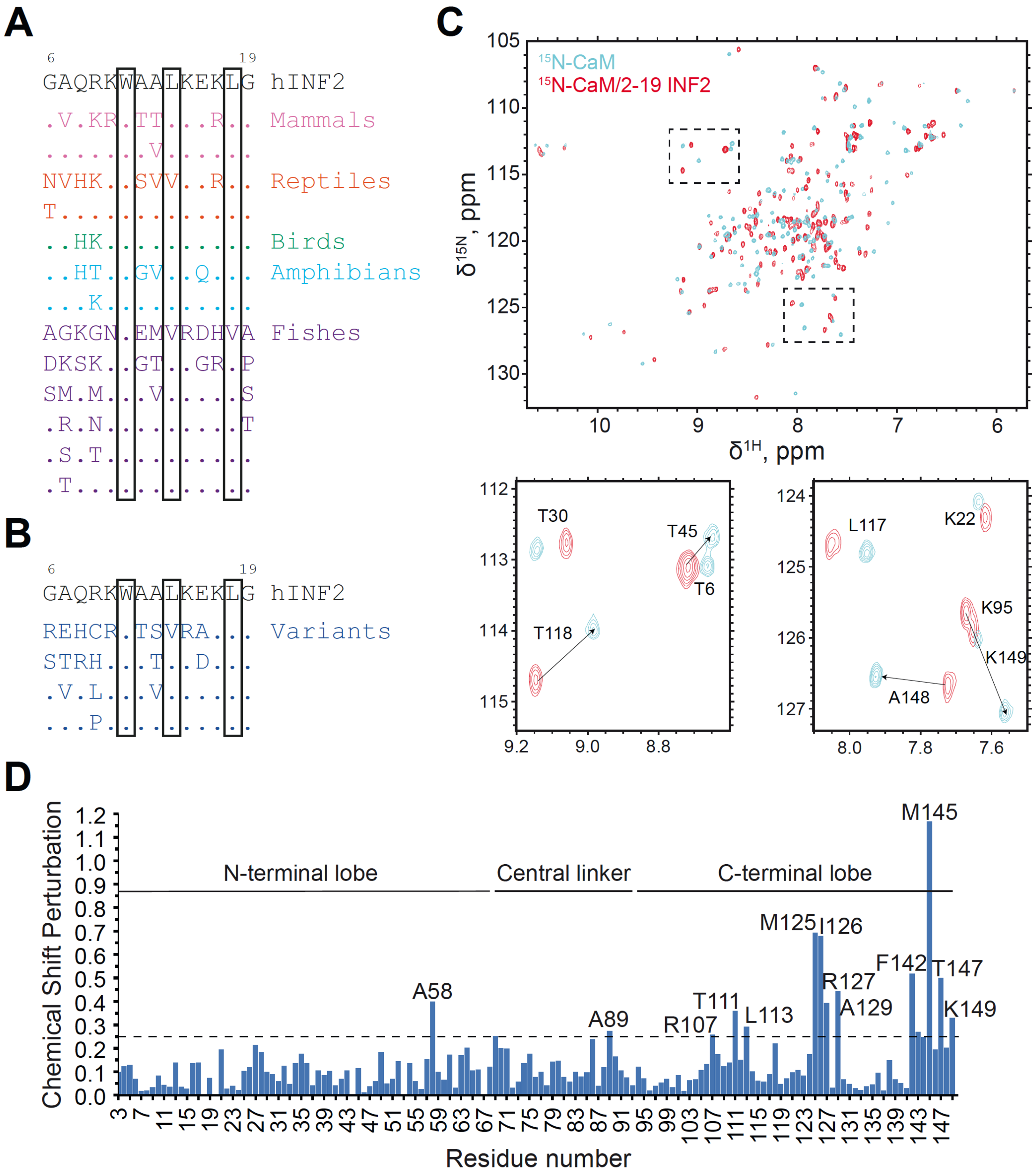

Figure S4. **Conservation of the CaM-binding motif of INF2 in vertebrates**. (**A**) Compilation of the amino acid replacements of the human 6-19 INF2 peptide found in more than 300 vertebrates. (**B**) Compilation of the amino acid replacements of the 6-19 INF2 peptide in the human population. (**C**) Overlay of the 2D [^{1}H–^{15}N]-HSQC spectrum of the free ^{15}N-CaM (in black) and ^{15}N-CaM/2-19 INF2 at an 1:1 ratio (in red). An enlargement of the boxed regions is shown. (**D**) Bar plot of the averaged ^{1}H and ^{15}N chemical shift perturbations ($\Delta\delta^{av} = \{[(\Delta\delta_{1H})^2 + (\Delta\delta_{15N}/5)^2]/2\}^{1/2}$, ppm) as a function of the residue number of CaM. The horizontal line indicates $\Delta\delta^{av}$ = 0.25 ppm, which represents the threshold. The residues above this value are indicated.

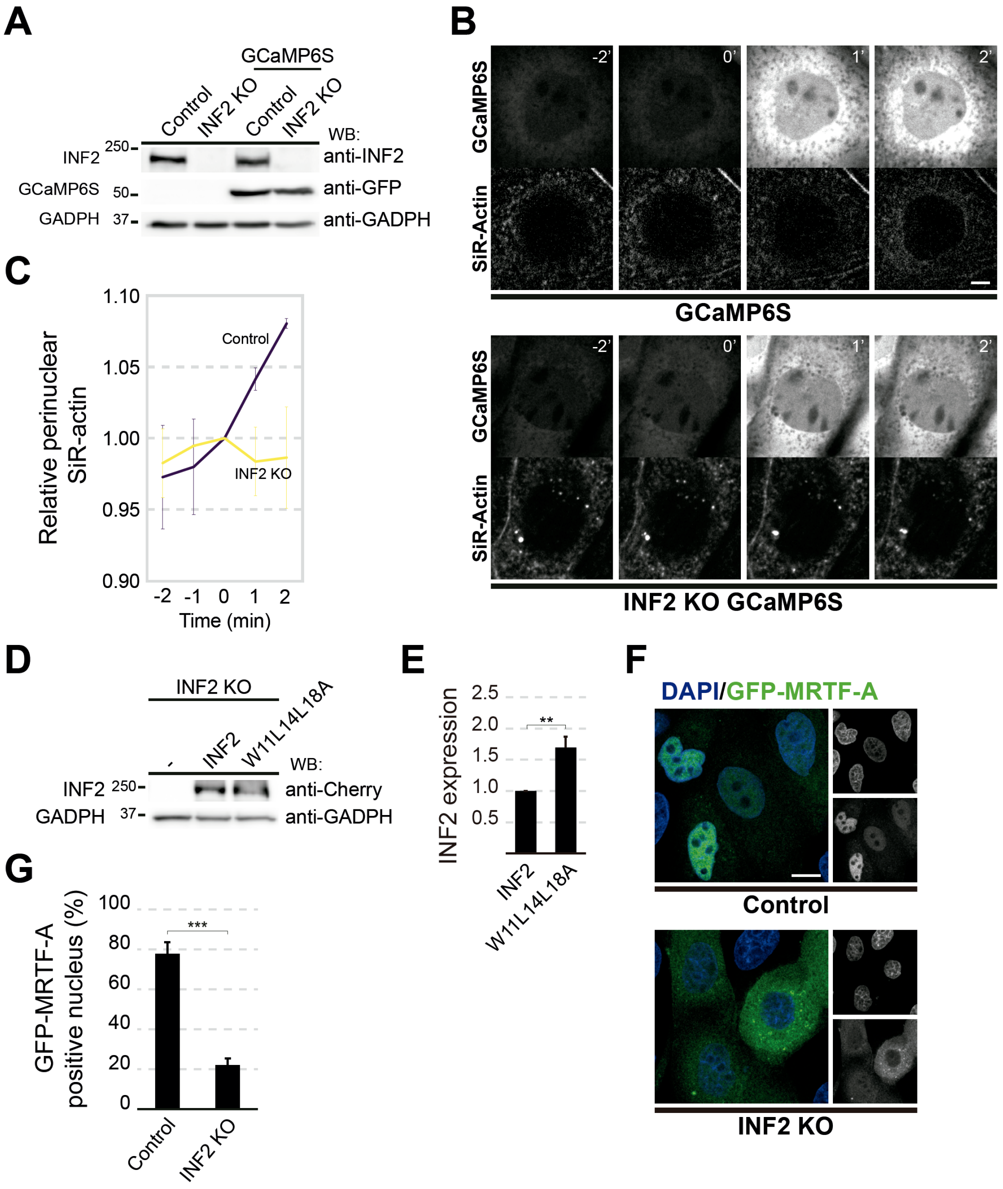

Figure S5. **INF2 MDCK KO cells are defective in actin polymerization and MRTF-A translocation to the nucleus**. (**A**) Control cells and INF2 KO cells expressing or not GCaMP6S were analyzed by immunoblotting with anti-INF2 antibodies to confirm the absence of INF2 in INF2 KO cells and with anti-GFP antibodies to analyze the levels of GCaMP6S. GADPH was used as a loading control. (**B**, **C**) Control cells and INF2 KO cells expressing GCaMP6S were incubated with SiR-actin to visualize F-actin. Cells were treated with A23187 (t = 0 min) and analyzed by videomicroscopy before and after treatment. Note that, although the staining of F-actin with SiR-Actin in live cells was poorer than that obtained with fluorescent phalloidin in fixed cells, it was useful to detect the increase of F-actin after A23187 stimulation (B). (C) Kinetics of the perinuclear content of F-actin as measured by SiR-actin fluorescence relative to that of cells at t = 0 min. Data represent the mean and SD of three independent experiments ($\geq$ 30 cells were analyzed; three independent experiments). (**D**, **E**) The expression of exogenous INF2-1 and INF2-1 W11L14L18A in INF2 KO cells was analyzed with anti-Cherry antibodies. The levels of GAPDH were taken as a loading control (D). The graph shows the expression of exogenous INF2-1 W11L14L18A relative to that of exogenous INF2-1 (E). Three independent experiments; **, $p < 0.01$. (**F**, **G**) Representative images of control cells and INF2 KO cells transiently expressing GFP-MRTF-A after treatment with A23187 (F). (G) Histogram of percentage of cells with GFP-MRTF-A in the nucleus. The mean and SD are shown (> 350 cells were examined; three independent experiments; ***, $p < 0.001$).

Table S1. Summary of structural statistical parameters for the ensembles of the 20 lowest target function conformers calculated for peptides INF2-2-19 and INF2-2-36 in 30 %TFE at pH 5.5.

**Peptide**	**INF2-2-19**	**INF2-2-36**
**Number of distance restraints**		
Intraresidue & sequential (i – j ≤ 1)	74	166
Medium range (1 < |i – j| < 5)	23	28
Long range (|i – j| ≥ 5)	0	0
Total number	97	194
Averaged total number per residue	5.4	5.5
**Number of dihedral angle constraints**		
Number of restricted ϕ angles	16	29
Number of restricted ψ angles	15	27
Total number	31	56
**Pairwise RMSD (Å)**		
**All residues (excluding N- and C-terminal)**	3-18	3-35
Backbone atoms	0.7±0.2	6.9±2.7
All heavy atoms	1.8±0.3	8.1±2.5
**N-terminal helix 1**	3-17	3-17
Backbone atoms	0.6±0.2	0.6±0.2
All heavy atoms	1.8±0.3	1.6±0.3
**Helix 2**	---	25-31
Backbone atoms	---	0.4±0.2
All heavy atoms	---	1.3±0.2
**Ramachandran plot (%)**		
Most favoured regions	98.3	89.8
Additionally allowed regions	1.7	10.2
Generously allowed regions	0.0	0.0
Disallowed regions	0.0	0.0

Table S2. List of NMR experiments acquired for ^{15}N-CaM and $^{15}N,^{13}C$-CaM

	Number of points	Spectral Width (ppm)	Number of scans	Time	NUS
Sequential Assignment of free $^{15}N,^{13}C$-CaM (25°C)					
2D ^{1}H-^{15}N HSQC	2k x 256	12(^{1}HN) x 29(^{15}N)	4	20min	--
3D HNCO	2k x 56 x 96	12(^{1}HN) x 29(^{15}N) x 14(^{13}CO)	4	7.2h	--
3D HNcaCO	2k x 56 x 96	12(^{1}HN) x 29(^{15}N) x 14(^{13}CO)	8	14.8h	--
3D HNCA	2k x 56 x 80	12(^{1}HN) x 29(^{15}N) x 30(^{13}C)	4	7.2h	--
3D HNcoCA	2k x 56 x 80	12(^{1}HN) x 29(^{15}N) x 30(^{13}C)	8	17.8h	--
3D CBCAcoNH	2k x 56 x 96	12(^{1}HN) x 29(^{15}N) x 80(^{13}C)	8	15h	--
3D HNCACB	2k x 56 x 80	12(^{1}HN) x 29(^{15}N) x 80(^{13}C)	24	36.5h	--
Sequential Assignment of $^{15}N,^{13}C$-CaM/INF2-2-19 at ratio 1:2 (25°C)					
2D ^{1}H-^{15}N HSQC	2k x 128	12(^{1}HN) x 29(^{15}N)	2	5min	--
3D HNCO	2k x 56 x 96	12(^{1}HN) x 29(^{15}N) x 14(^{13}CO)	4	1.5h	25%
3D HNCA	2k x 56 x 80	12(^{1}HN) x 29(^{15}N) x 30(^{13}C)	4	3.0h	50%
3D CBCAcoNH	2k x 56 x 96	12(^{1}HN) x 29(^{15}N) x 80(^{13}C)	8	6.5h	45%
3D HNCACB	2k x 56 x 80	12(^{1}HN) x 29(^{15}N) x 80(^{13}C)	24	14.3h	40%
